# Intron Derived Size Polymorphism in the Mitochondrial Genomes of Closely Related *Chrysoporthe* Species

**DOI:** 10.1371/journal.pone.0156104

**Published:** 2016-06-06

**Authors:** Aquillah Mumo Kanzi, Brenda Diana Wingfield, Emma Theodora Steenkamp, Sanushka Naidoo, Nicolaas Albertus van der Merwe

**Affiliations:** 1 Department of Genetics, Forestry and Agricultural Biotechnology Institute (FABI), University of Pretoria, Private bag x20, Pretoria, 0028, South Africa; 2 Department of Microbiology and Plant Pathology, Forestry and Agricultural Biotechnology Institute (FABI), University of Pretoria, Private bag x20, Pretoria, 0028, South Africa; University of California, UNITED STATES

## Abstract

In this study, the complete mitochondrial (mt) genomes of *Chrysoporthe austroafricana* (190,834 bp), *C*. *cubensis* (89,084 bp) *and C*. *deuterocubensis* (124,412 bp) were determined. Additionally, the mitochondrial genome of another member of the *Cryphonectriaceae*, namely *Cryphonectria parasitica* (158,902 bp), was retrieved and annotated for comparative purposes. These genomes showed high levels of synteny, especially in regions including genes involved in oxidative phosphorylation and electron transfer, unique open reading frames (uORFs), ribosomal RNAs (rRNAs) and transfer RNAs (tRNAs), as well as intron positions. Comparative analyses revealed signatures of duplication events, intron number and length variation, and varying intronic ORFs which highlighted the genetic diversity of mt genomes among the *Cryphonectriaceae*. These mt genomes showed remarkable size polymorphism. The size polymorphism in the mt genomes of these closely related *Chrysoporthe* species was attributed to the varying number and length of introns, coding sequences and to a lesser extent, intergenic sequences. Compared to publicly available fungal mt genomes, the *C*. *austroafricana* mt genome is the second largest in the Ascomycetes thus far.

## Introduction

*Chrysoporthe* species are economically important pathogens of *Eucalyptus* species and other *Myrtales* [1, 2]. Species in this genus include *Chrysoporthe austroafricana* [3], *Chrysoporthe cubensis* [4, 5], *Chrysoporthe inopina* [6], *Chrysoporthe hodgesiana* [1], *Chrysoporthe doradensis* [7], *Chrysoporthe deuterocubensis* [8], *Chrysoporthe zambiensis* and *Chrysoporthe syzigiicola* [9]. These species form part of the family *Cryphonectriaceae*, which also includes *Cryphonectria* [1, 2, 8, 10]. Although the molecular data used in these studies have helped to clarify the species boundaries in *Chrysoporthe*, the relationship among the members of this genus remains largely unresolved [8, 9].

Mitochondrial (mt) sequences have been used to determine and/or confirm phylogenetic relationships, especially in cases where nuclear genes have not accumulated sufficient phylogenetic signal, to clarify conflicting phylogenies [11, 12]. This is because mt genomes contain marker genes that are particularly suited to studies in evolutionary biology and systematics [13–15]. Additionally, due to the relatively small sizes of mt genomes, whole genome analysis can provide a large set of homologous genes that can be directly compared [16]. The advantages of using mt genes for phylogenetic inference are numerous–their evolution is largely independent from nuclear genes, they are uniparentally inherited, they display a uniform genetic background, and they have limited rates of recombination [17]. Furthermore, due to an increase in whole genome sequencing projects, there are a large number of complete mt genomes in the public domain (http://www.ncbi.nlm.nih.gov/genome/browse/?report=5). The availability of these genomic resources has enhanced their usage in evolutionary biology [12, 18–22].

Fungal mt genomes are circular A+T rich sequences that usually contain a set of 14 conserved genes that encode proteins involved in oxidative phosphorylation (OXPHOS) and electron transport [16, 23]. These include three ATP synthase subunits (*atp6*, *atp8* and *atp9*), three subunits of cytochrome oxidase (*cox1*, *cox2* and *cox3*), apocytochrome b (*cob*) and seven subunits of the nicotinamide adenine dinucleotide ubiquinone oxidoreductase subunits (*nad1*, *nad2*, *nad3*, *nad4*, *nad4L*, *nad5* and *nad6*). Untranslated genes include the small subunit ribosomal RNA (*rns*) and the large subunit ribosomal RNA (*rnl*), as well as a varying number of transfer-RNA (tRNA) genes [16, 17]. Additionally, mitochondrial genomes are known to harbour intergenic ORFs, some of which are unique to each genome (uORFs), while others are mobile ORFs encoding endonucleases [24–27]. Linear or circular plasmids, plasmid-like and virus-like elements have also been reported from mitochondria and mt genomes [28–31].

The diversity of mt genomes is evident among Ascomycetes, where the smallest mt genome is 18,512 bp for *Hanseniaspora uvarum* [32] and the largest is 203,051 bp for *Sclerotinia borealis* [33]. These size differences appear to be directly correlated to the numbers and sizes of introns, the presence of dispersed repetitive regions, and new genes introduced through horizontal transfer [25, 34–36]. The divergence of these mt genomes is also associated with rearrangements in gene order [15, 22] and the presence or absence of auto-replicating plasmids [30].

Fungal mt introns encode various homing endonuclease genes (HEGs) [31, 37–39]. These intron-enclosed genes have homing endonuclease (HE) activity, thus are able to move or self-integrate (homing) themselves into specific locations in the genome [31, 38, 39]. The introns occurring in mt genomes are divided into two groups, namely groups I and II, both of which encode HEGs with signature LAGLIDADG or GIY-YIG domain motifs [40, 41]. These introns generally seem to affect the mt genome structure by influencing variations in genome size, gene order, gene duplications, and the introduction of exogenous genes via horizontal gene transfer [15, 33, 35, 36].

Despite the increasing number, and potential applications, of mtDNA in systems biology and evolutionary studies [14, 42, 43], the mt genome sequences of *Chrysoporthe* species have not yet been determined. The aim of this study was therefore, to provide insights into the structure, organization and source of variability in the mt genomes of these closely related species. Additionally, availability of these genomes also provides invaluable resources for evolutionary studies. For this reason, the complete mt genomes of *C*. *austroafricana*, *C*. *cubensis* and *C*. *deuterocubensis* were assembled and annotated. Also, for comparative purposes, the mt genome for *C*. *parasitica* was fully annotated. Comparisons were focused on genome organization, gene order and gene content of these four mt genomes.

## Materials and Methods

### Genome sequencing and assembly

The mt genome of *C*. *austroafricana* was sequenced as part of a whole genome sequencing project [44, 45]. Additionally, *C*. *cubensis* and *C*. *deuterocubensis* mt genome sequences were obtained from subsequent whole genome sequencing projects (Wingfield *et al*. 2015, submitted). The *C*. *parasitica* mt genome sequence was obtained from the Joint Genome Institute (JGI) database (http://genome.jgi.doe.gov/Crypa2/Crypa2.download.html). After assembly of the *C*. *austroafricana*, *C*. *cubensis* and *C*. *deuterocubensis* nuclear genomes, the mt genome of *C*. *parasitica* was used to identify conserved mt gene regions by reference assembly in CLC Genomics Workbench v 7.0.1 (CLC Bio, Arhus, Denmark). Identified mt sequences were further confirmed by BLASTn [46] searches against non-redundant nucleotide sequences in the NCBI GenBank database. The confirmed sequences were then used as seeds for iterative contig extension using MITObim v 1.7 beta [47] and PRICE v 1.2 [48] software. Extended contigs were aligned using the multiple sequence alignment program MAFFT v 7.182 [49] where contigs with more than 500 bp of overlapping sequences were merged into larger contigs. After confirming the presence of all the conserved mt genes in the assembled contigs, contigs that displayed overlaps at both ends were used to circularize the mt genomes. To verify the assemblies, raw paired-end sequence reads were mapped to the circularized genomes using CLC Genomics Workbench.

### Mitochondrial genome annotation

The mt genomes of *C*. *austroafricana*, *C*. *cubensis* and *C*. *deuterocubensis* were annotated using the MFANNOT tool (http://megasun.bch.umontreal.ca/cgi-bin/mfannot/mfannotInterface.pl) with default settings. The *C*. *parasitica* mt genome was annotated via BLASTn searches using previously published *C*. *parasitica* mt genes deposited in GenBank, and completed using the MFANNOT tool. Additionally, open reading frames (ORFs) were predicted in CLC Genomics Workbench v 7.0.1 and compared to those predicted by MFANNOT. ORFs coding for hypothetical proteins were identified by BLASTP against the NCBI with a cut-off of 50% sequence identity. Introns and tRNA genes were characterized using the online program RNAweasel (http://megasun.bch.umontreal/ca/RNAweasel). ORFs encoding HEGs were identified based on their conserved domains through similarity searches in the Pfam (http://pfam.xfam.org/) and InterPro (http://www.ebi.ac.uk/interpro/) protein domain databases, as well as the NCBI (BLASTP) [46].

### Sequence and phylogenetic analysis

Gene order among the four mt genomes was determined by comparing pairwise BLASTN results using genoPlotR [50]. Further, similarity information was obtained from pairwise sequence alignments using MAFFT. Codon usage was analysed using the web-based Sequence Manipulation Suite (http://www.bioinformatics.org/sms2/codon_usage.html) with the fungal mt genetic code 4. To identify the presence of direct sequence repeats and inverted sequence repeats in intergenic regions of the mt genomes, REPFIND (http://zlab.bu.edu/repfind/) and EMBOSS [51] were used respectively.

All phylogenetic analyses in this study were performed using maximum likelihood (ML) method implemented in RAxML [52]. Sequence alignments were generated from MAFFT were trimmed of poorly aligned regions using trimAL [53]. The best models of evolution were selected using jModelTest v 2.1.5 [54] for nucleotide sequences and ProtTest version 3.4 [55] for amino acid sequences. To determine the confidence of the recovered nodes, bootstrap analysis was performed with 1000 resampling steps.

## Results

### Mitochondrial genome structure and organization

**Fig 1:**
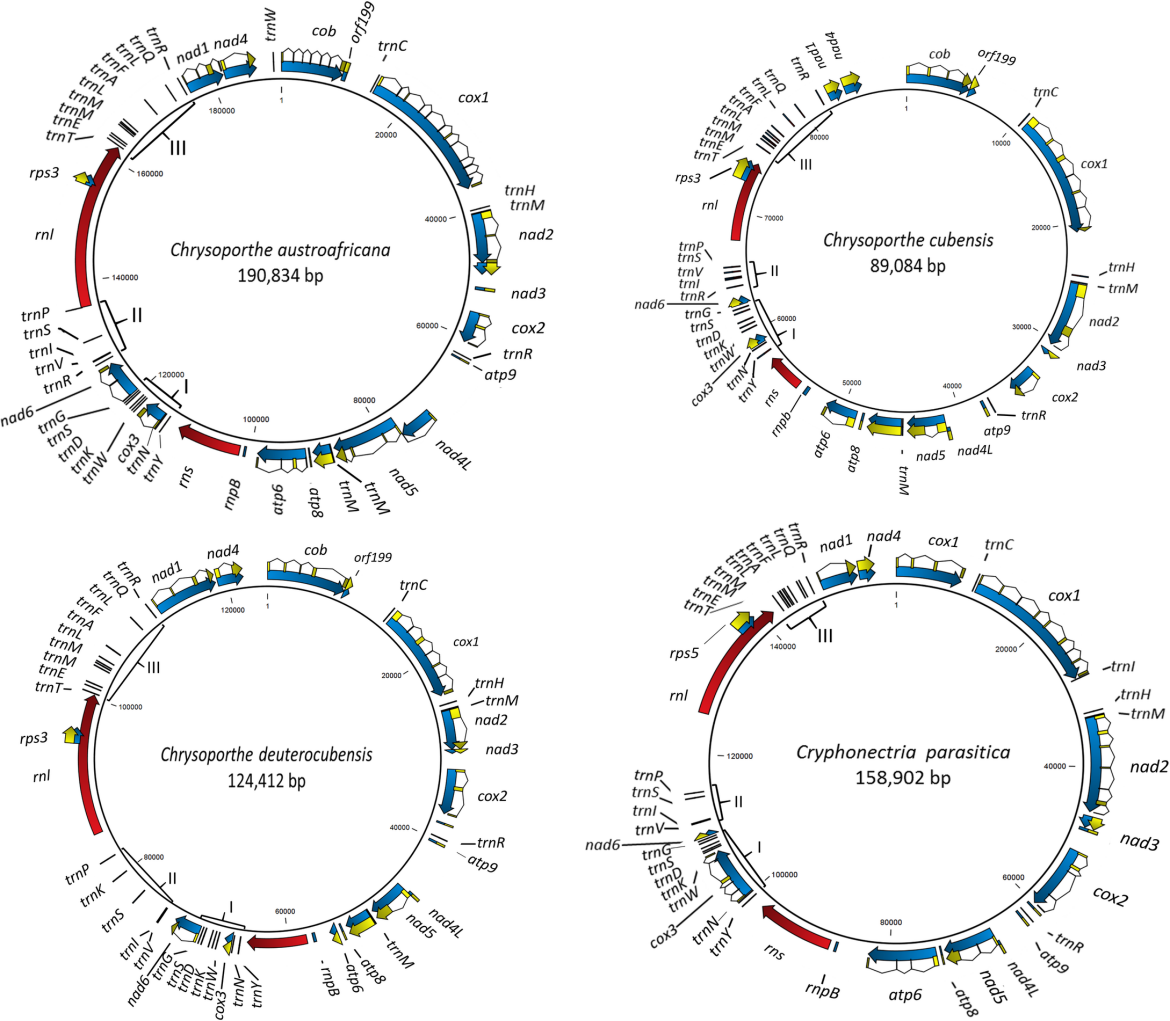
Physical maps of the mitochondrial (mt) genomes of *Chrysoporthe austroafricana*, *C*. *cubensis*, *C*. *deuterocubensis* and *C*. *parasitica* mitochondrial (mt) genomes. The 14 genes involved in oxidative phosphorylation and electron transport are shown. Blue arrowed lines indicate full length genes and direction of transcription. Yellow blocks connected by lines indicate the coding sequences (CS), red arrowed lines show the large and small subunit ribosomal RNAs, and black lines indicate transfer RNA (tRNA) genes. Curly brackets labelled I, II and III indicate the major tRNA clusters.

**Fig 2:**
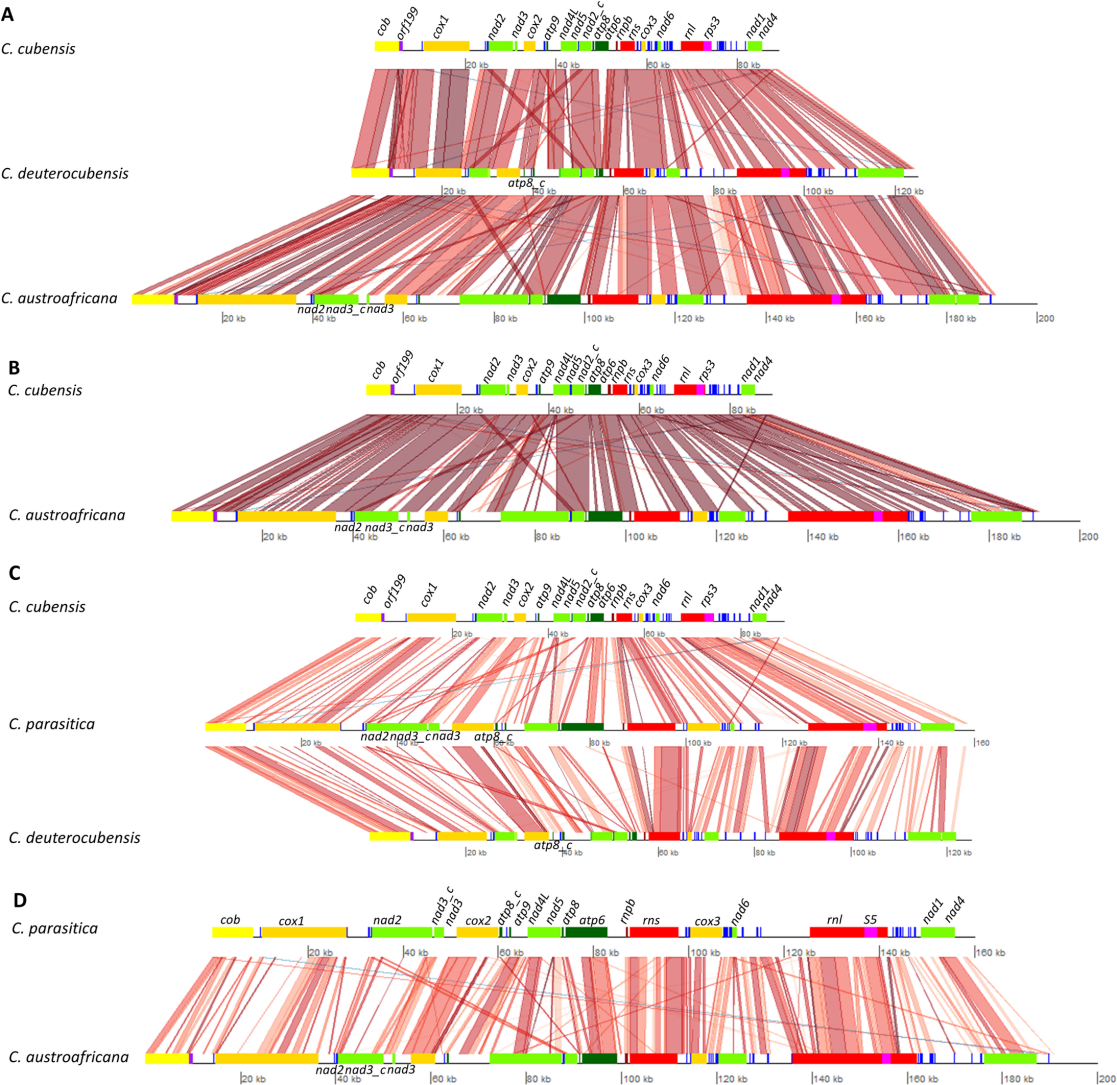
Mitochondrial genome synteny maps. **A**. Synteny comparisons between *C*. *cubensis*, *C*. *deuterocubensis* and *C*. *austroafricana*. **B**. Synteny comparison between *C*. *cubensis* and *C*. *austroafricana*. **C**. Comparisons between *C*. *cubensis*, *C*. *parasitica* and *C*. *deuterocubensis*. **D**. Comparison between *C*. *parasitica* and *C*. *austroafricana*. The Blast2Seq feature in NCBI was used to identify syntenic blocks using BLASTN. Alignments of length of greater than 100 bp and e-value > = 0.00005 are shown. Yellow boxes represent *cox* genes, gold; *cob*, lawn green; *nad* genes, green; *atp* synthase genes, blue; *tRNAs*, red; ribosomal RNAs and purple; *rps3*. Synteny graphs were generated using GenoPlotR.

The mt genomes of *C*. *austroafricana* (GenBank accession no. KT380883), *C*. *cubensis* (GenBank accession no. KT380885), *C*. *deuterocubensis* (GenBank accession no. KT380884) and *C*. *parasitica* (Genbank accession no. KT428651) are circular mtDNA molecules of 190,834 bp, 89,084 bp, 124,412 bp, and 158,902 bp in size, respectively (Fig 1). The *C*. *parasitica* mt genome had the highest G+C content of 32.04%, followed by *C*. *austroafricana* with 30.25%, 29.98% for *C*. *deuterocubensis*, and 27.91% for *C*. *cubensis*. Pairwise whole mt genome sequence alignments revealed that *C*. *austroafricana* shared 41%, 46% and 55% sequence identity with *C*. *parasitica*, *C*. *cubensis* and *C*. *deuterocubensis*, respectively. The *C*. *cubensis* mt genome respectively shared 56% and 32% sequence identity with *C*. *deuterocubensis* and *C*. *parasitica*, while *C*. *deuterocubensis* and *C*. *parasitica* shared 35% sequence identity. Pairwise BLAST comparisons of these mt genomes identified conserved and syntenic blocks of sequences especially between OXPHOS genes (Fig 2). No sequence repeats of notable sizes were identified in any of the four mt genomes.

All fourteen expected mt protein coding genes (*atp6*, *atp8*, *atp9*, *cox1*, *cox2*, *cox3*, *cob*, *nad1*, *nad2*, *nad3*, *nad4*, *nad4L*, *nad5* and *nad6*) could be identified. Additionally, the small subunit RNA (*rns*), large subunit RNA (*rnl*) and RNA subunit of mt RNase P (*rnpB*) genes were identified in all four species (Fig 1). BLAST searches for other mitochondrial *rnpb* genes in GenBank database did not yield many hits however, six *rnpb* genes were identified by manually searching for annotated *rnpb* genes in ascomycete mt genomes deposited in GenBank. These genes were located in similar positions in the mt genomes where present (S1 Fig). ORFs homologous to *rps3* gene, orf540, orf551 and orf551 were identified in mt genomes of *C*. *austroafricana*, *C*. *cubensis* and *C*. *deuterocubensis*, respectively. These ORFs encoded a protein that shared 88% amino acid sequence identity with the putative S5 ribosomal protein/maturase fusion protein identified in the *C*. *parasitica* mt genome [56]. However, from the conserved domain and BLAST analysis, these ORFs harbour a HEG, a feature that was observed in *rps3* genes of other fungi (S2 Fig). Phylogenetic analysis grouped orf540, orf551 and orf551 with rps3/HEG fusion proteins (S2 Fig and S1 Table). Expression analysis based on available RNA-Seq data from *C*. *austroafricana* revealed that all these genes were actively expressed in minimal and complete media growth conditions (S3 Fig and Mangwanda *et al*., submitted).

All of the conserved mt genes were encoded on a single strand and were in the same direction and order (Fig 1). Although gene order was conserved among these four mt genomes, variations could be observed when compared to mt genomes of other filamentous fungi. However, there was conservation in the order of particular genes that are known to occur adjacent to each other, such as *nad2-nad3*, *atp6-atp8*, *cob-tRNA-Cys-cox1*, *nad4L-nad5* and *nad1-nad4* among *Sordariomycetes* [15, 22]. One notable difference was observed in the *C*. *austroafricana* mt genome, where the *nad4L* gene had an intron. This intron contained two ORFs, both encoding for HEGs with LAGLIDADG domains. Overall, the genes encoded in these mt genomes showed high levels of synteny (Fig 2).

A total of 28, 27, 27 and 26 tRNA genes (Table 1) coding for all amino acids were predicted in the mt genomes of *C*. *austroafricana*, *C*. *cubensis*, *C*. *deuterocubensis* and *C*. *parasitica*, respectively. All *Chrysoporthe* species had four *tRNA-Met* genes compared to three in *C*. *parasitica*. The *tRNA* genes in the four mt genomes were clustered, as is the case in most mt genomes of *Pezizomycotina* [15]. Three major clusters of *tRNA* genes were identified. The first cluster was between the *rns* and *nad6* genes and contained genes encoding *tRNA-Try*, *tRNA-Asn*, *tRNA-Trp*, *tRNA-Lys*, *tRNA-Asp*, *tRNA-Ser* and *tRNA-Gly* (Fig 1). Cluster 2, which included genes for *tRNA-Arg*, *tRNA-Val*, *tRNA-Ile*, *tRNA-Ser*, and *tRNA-Pro*, was located between the *nad6* and *rnl genes* (Fig 1). This cluster in the *C*. *deuterocubensis* mt genome did not have the *tRNA-Arg* gene, but instead, a *tRNA-Lys* gene was inserted between the *tRNA-Ser* and *tRNA-Pro* genes. Likewise, the *tRNA-Arg* gene was absent in the second *tRNA* cluster of four *tRNA* genes of the *C*. *parasitica* mt genome. The third cluster was the largest of the three, and had 10 *tRNA* genes located between the *rnl* and *nad1* (Fig 1). These were the *tRNA-Thr*, *tRNA-Glu*, *tRNA-Met*, *tRNA-Met*, *tRNA-Leu*, *tRNA-Ala*, *tRNA-Phe*, *tRNA-Leu*, *tRNA-Gln* and *tRNA-Arg* genes. Only one *tRNA-Ile* gene was present in all three *Chrysoporthe* species, while two non-adjacent copies of this gene were observed in the *C*. *parasitica* mt genome (Fig 1 and S2 Table). Two *tRNA-Gly* genes were predicted in *C*. *austroafricana*, while only one was predicted in the other mt genomes. Likewise, two *tRNA-Lys* genes were predicted in the *C*. *deuterocubensis* mt genome, compared to one in the other mt genomes (Fig 1 and S2 Table).

**Table 1:** Mitochondrial genome features. Number of genes, open reading frames (ORFs), rRNA, tRNAs and introns of the mitochondrial genomes of *Chrysoporthe austroafricana*, *C*. *cubensis*, *C*. *deuterocubensis* and *Cryphonectria parasitica*. Intergenic ORFs were considered uORFs if no HEG domains or significant BLAST hits could be identified.

Species	Intergenic ORFs	Intronic ORFs	uORFs	rRNAs	tRNAs	GrpI/II Introns
*C*. *austroafricana*	21	42	8	2	28	32/3
*C*. *cubensis*	7	11	5	2	27	14/0
*C*. *deuterocubensis*	17	20	10	2	27	19/0
*C*. *parasitica*	14	34	6	2	26	27/5

### Codon usage

The codon usage frequencies for all the conserved protein coding mitochondrial genes were calculated from the respective CS (S2 Table). The start codon ATG was favoured by most CS across all four species. GTG was favoured as the start codon for *nad4* in all four species, while *cox1* favoured TTG. In the *nad6* genes, *C*. *parasitica* had TTG as the start codon, in contrast to ATG in the *Chrysoporthe* species. The most frequently used stop codon was TAA, apart from *cox1* and *nad1* in all species, which had TAG as the stop codon. The TAG stop codon was also observed in *atp9*, *nad5* and *nad4* CS of *C*. *austroafricana*, *C*. *deuterocubensis* and *C*. *parasitica*, respectively. Among the intronic ORFs encoding proteins the preferred start codon was ATG, while TAA was the most frequent stop codon. Similarly, ATG was the start codon for all the intergenic ORFs encoding proteins in all species while TAA was the stop codon. Nonetheless, a few ORFs (4, 3, 2 and 7) had TAG as the stop codon *C*. *austroafricana*, *C*. *deuterocubensis* and *C*. *parasitica*, respectively. The most frequently used codons in the OXPHOS genes were those that code for hydrophobic amino acids, including Leu (TTA), Ile (ATA) and Phe (TTT), accounting for approximately 10%, 6% and 5% of all codons in all four species (S2 Table). The codon usage of intergenic ORFs, intronic ORFs, and duplicated genes did not differ from that of the conserved mt genes (data not shown).

The *C*. *austroafricana* and *C*. *cubensis* mt genomes shared three *tRNA-Arg* genes with similar anti-codons (TCT, TCT and ACG), unlike in *C*. *deuterocubensis* and *C*. *parasitica* where only two *tRNA-Arg* genes, with anticodons TCT and ACG, were observed. Most anticodons in the tRNA genes had a T or A nucleotide at the third position. Some amino acid codons in the mt genomes lacked corresponding tRNA genes (S2 Table).

### Gene duplication

**Fig 3:**
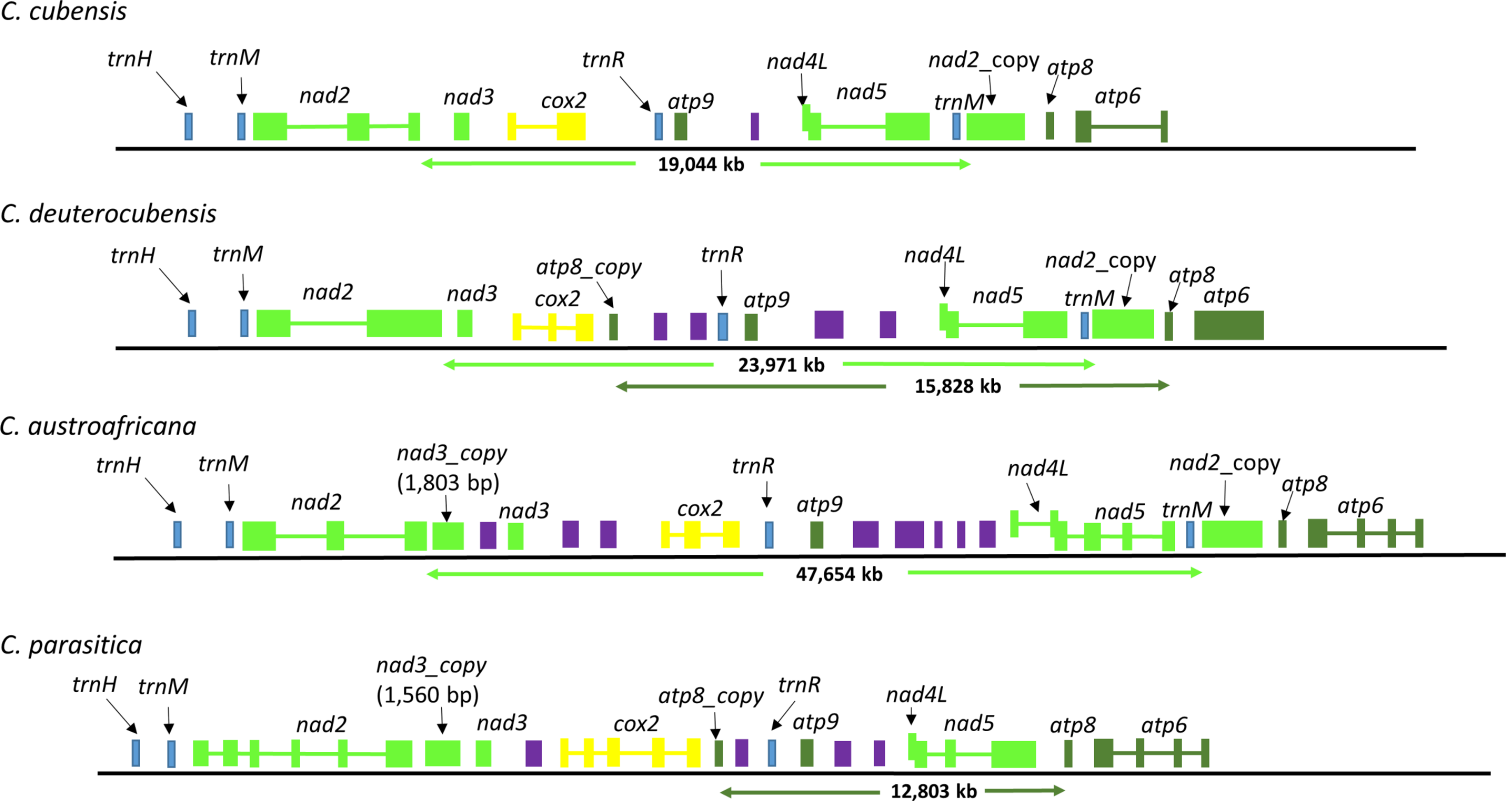
Organization of genes around the *nad2*, *nad3 and atp8* genes showing partially duplicated sequences. Dark green, light green, olive green, red, yellow, light blue and purple boxes represent the *nad2*, *nad3* and *nad5*, *cox2*, *atp9*, *tRNAs* genes and various open reading frames (ORFs), respectively. The duplicate genes are denoted as *nad2_copy*, *atp8_copy* and *nad3_*copy.

The mt genomes of the three *Chrysoporthe* species contained some genomic regions that were duplicated (Fig 3). One of these duplicated regions resulted in an ORF that appears to be partially, homologous to the *nad2* gene. This ORF, located between *nad5* and *atp8*, was 2,718 bp in size in all three mt genomes and shared between 97–99% pairwise DNA sequence identities between the three species. Both *nad2* and the ORF with partially duplicated *nad2* sequences were flanked by a stretch of homologous sequences including a *tRNA-M* gene in the upstream direction (Fig 3). This stretch was 154 bp, 153 bp, 150 bp and 151 bp, 153 bp, and 163 bp in *C*. *austroafricana*, *C*. *cubensis* and *C*. *deuterocubensis* respectively. The region between *nad2* and this ORF was 19,044 bp, 23,971 bp and 47,654 bp in *C*. *cubensis*, *C*. *deuterocubensis* and *C*. *cubensis* respectively (Fig 3). Sequence alignment of *nad2* gene and the duplicated region revealed up to 96% sequence identity over a region spanning 814 bp, 816 bp and 816 bp covering part exon1 of *nad2* gene in *C*. *austroafricana*, *C*. *cubensis* and *C*. *deuterocubensis*, respectively.

Two ORFs of were annotated as *atp8* genes by MFANNOT in the *C*. *deuterocubensis* and *C*. *parasitica* mt genomes but only one copy in *C*. *cubensis* and *C austroafricana*. In the mt genomes of ascomycetes, *atp8* gene is usually adjacent to the *atp6* gene[15]. The additional ORFs located between the *cox2* and *atp9* genes of length 159 bp and 219 in *C*. *deuterocubensis* and *C*. *parasitica* respectively (Fig 3 and S4 Fig). The endogenous *atp8* genes which were 165 bp and 168 bp long shared 93% and 100% sequence identity over a stretch of 130 bp and 147 bp respectively in *C*. *deuterocubensis* and *C*. *parasitica*. BLASTN comparison of these duplicate ORFs revealed 83% sequence identity over a 146 bp alignment. Analysis of sequences flanking both copies of the duplicated genes did not identify homologous sequences. The distance between the duplicate ORFs and *atp8* gene was 15,828 bp in *C*. *deuterocubensis* and 12,803 bp *C*. *parasitica*.

ORFs of 1,803 bp and 1,560 bp with sequences partially similar to the *nad3* genes *C*. *austroafricana* and *C*. *parasitica* were identified (Fig 3 and S4 Fig). Comparative nucleotide sequence analysis of these ORFs to the respective *nad3* genes revealed a homologous region of 368 bp and 160 bp in the 5’ region both sharing 82% sequence identity in *C*. *austroafricana* and *C*. *parasitica* respectively. The 3’ region of both ORFs had sequences coding for GIY-YIG endonuclease domain. Pairwise comparison of the amino acid sequence product of these ORFs that were homologous to *nad3* in *C*. *austroafricana* and *C*. *parasitica*, showed 48% sequence identity. 2,061 bp separated the ORFs with partially duplicated *nad3* sequences and the endogenous *nad3* genes was and 98 bp in *C*. *austroafricana* and *C*. *parasitica* respectively.

### Introns and intron-encoded ORFs

**Fig 4:**
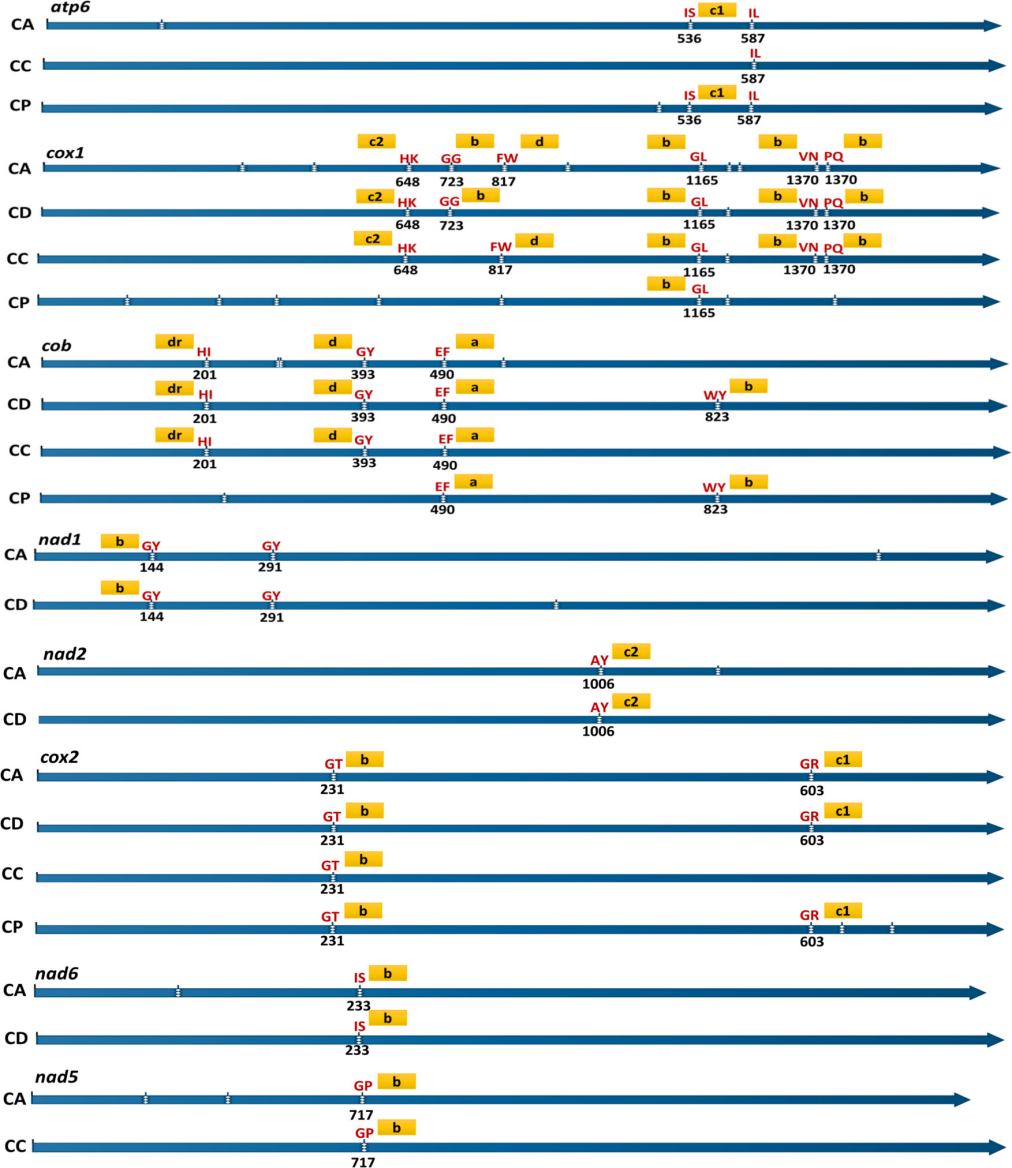
Conserved intron insertion position and intron type identified within the *atp6*, *cox1*, *cob*, *nad1*, *nad2*, *cox2*, *nad6*, *and nad5* of *C*. *austroafricana*, *C*. *cubensis*, *C*. *deuterocubensis* and *C*. *parasitica*. Blue lines with arrow heads represent genes compared. White vertical breaks within the blue lines represent intron position with the insertion position is shown below it. Intron type is indicated by letters in gold boxes. Amino acid residues bordering the exonic sequences are shown in red single letter amino acid notation. ***A*;**group 1A introns, ***b***; group 1B, ***c1***; group 1C1, ***c2***; group 1C2, ***d***; group 1D, ***dr***; group I-derived.

Both group I and II introns [37] were identified in *C*. *austroafricana* and *C*. *parasitica* mt genomes, while only group I introns were identified in *C*. *cubensis* and *C*. *deuterocubensis* mt genomes (Tables 1 and 2). BLASTN analysis of introns from the four mt genomes revealed introns with sequence identities over 70% and alignment coverage over 50% (data not shown). However, only a few orthologous introns were identified. For example, intron 1 of *cob* in *C*. *austroafricana* and *C*. *cubensis* shared 100% sequence identity and had the same insertion point, but the same intron in the *cob* of *C*. *deuterocubensis* only showed 95% sequence identity with the *C*. *austroafricana* and *C*. *cubensis* introns (Fig 4). Intron 1 of the *cob* gene in *C*. *parasitica* shared up to 80% sequence identity with that of *C*. *austroafricana*, *C*. *cubensis and C*. *deuterocubensis* but had a different insertion point in (Fig 4) thus was not considered orthologous. Intron 5 of *C*. *austroafricana cob* had the same insertion point in *C*. *cubensis* (intron 3), *C*. *deuterocubensis* (intron 3) and *C*. *parasitica* (intron 2). Overall, only 17 introns could be considered orthologous based on sequence identity and insertion point in the exonic sequence. Only three introns located in the *cox1*, *cob* and *cox2* genes were orthologous in all the four mt genomes analysed. Orthologous introns were mostly identified in the mt genomes of the closely related *Chrysoporthe* species compared to distantly related *C*. *parasitica*. BLAST analysis of the identified introns against NCBI GenBank database revealed sequence identities with sequences from other fungal mt genomes (S3 Table).

**Table 2:** Diversity of mitochondrial introns and intron encoded HEGs in the mt genomes of *C*. *austroafricana*, *C*. *cubensis*, *C*. *deuterocubensis* and *C*. *parasitica*. Lower case ***a*** group stands for group 1A introns, ***b*** group 1B, ***c1*** group 1C1, ***c2*** group 1C2, ***d*** group 1D, ***dr*** group 1-derived introns. Lower case *i* stands for intron and the numbers indicate intron positions in the respective mitochondrial gene. Superscript letters *l*, *g* and *rt* stand for intron encoded LAGLIDADG, GIY-YIG type HEGs and reverse transcriptase encoding ORFs respectively.

Intron type	site	*C*. *austroafricana*	*C*. *cubensis*	*C*. *deuterocubensis*	*C*. *parasitica*
Group1	*atp6*	*i1*_*c2*_^*ll*^, *i2*_*c2*_^*g*^, *i3*^*gg*^	*i1*^*g*^		*i2*_*c2*_^*g*^, *i3*_*c2*_^*gg*^
	*cox1*	*i1*_*b*_^*ll*^,*i2*_*b*_^*l*^,*i3*_*c2*_^*ll*^,*i4*_*b*_^*l*^,*i5*_*d*_^*gl*^,*i6*_*b*_^*l*^,*i7*_*b*_^*g*^,*i8*_*dr*_^*ll*^,*i9*_*b*_^*l*^,*i10*_*b*_^*g*^,*i11*_*b*_^*g*^	*i1*_*c2*_^*l*^,*i2*_*d*_^*l*^,*i3*_*b*_^*g*^, *i4*_*dr*_^*l*^,*i5*_*b*_^*g*^,*i6*^*g*^	*i1*_*c2*_^*l*^,*i2*_*b*_^*l*^,*i3*_*b*_^*g*^,*i4*_*dr*_^*l*^,*i5*_*b*_^*g*^,*i6*_*b*_^*g*^	*i2*_*b*_^*l*^,*i4*_*b*_^*g*^,*i5*^*gl*^,*i6*_*b*_^*g*^,*i7*_*dr*_^*l*^,*i8*_*b*_^*g*^
	*cox2*	*i1*_*b*_^*g*^, *i2*_*c1*_^*g*^	*i1*_*b*_^*g*^	*i1*^*g*^, *i2*_*c1*_^*g*^	*i1*_*b*_^*l*^, *i2*_*c1*_, *i3*^*l*^, *i4*_*c1*_^*g*^
	*cox3*	*i1*_*b*_^*ll*^			*i1*, *i2*_*a*_^*l*^, *i3*_*c2*,_*i4*_*c1*_^*g*^
	*cob*	*i1*_*dr*_^*l*^, *i4*_*d*_^*g*^, *i5*_*a*_^*l*^, *6*_*b*_^*l*^	*i1*_*dr*_^*l*^, *i2*_*d*_^*g*^, *i3*_*a*_^*l*^	*i1*_*dr*_^*l*^, *i2*^*g*^, *i3*_*a*_^*l*^, *i4*_*b*_^*gg*^	*i1*_*dr*_^*l*^, *i2*_*a*_^*l*^, *i3*_*b*_^*gg*^
	*nad1*	*i1*_*b*_^*g*^, *i2*^*l*^, *i3*		*i1*^*g*^_*b*_, *i2*^*ll*^, *i3*_*b*_,	*i1*_*c1*_
	*nad2*	*i1*_*c2*_, *i2*_*c2*_^*g*^	*i1*_*c2*,_ *i2*_*c2*_	*i1*_*c2*_	*i1*_*c2*_^*l*^, *i2*^*l*^, *i3*_*c2*_^*l*^, *i5*_*c2*_^*l*^, *i6*
	*nad4*	*i1*_*c2*_^*l*^		*i1*_*c2*_^*l*^	
	*nad4L*	*i1*_*c2*_^*ll*^			
	*nad5*	*i1*_*b*_^*l*^, *i2*^*lg*^,*i3*^*l*^	*i1*_*b*_^*l*^	*i1*_*d*_^*l*^	*i1*^*l*^, *i2*_*c2*_^*ll*^
	*nad6*	*i2*^*l*^		*i1*^*l*^	
Group 2	*cox1*				*i1*^*rt*^, *i3*^*rt*^
	*cob*	*i2*, *i3*^*l*^			
	*nad2*				*i4*^*rt*^
	*nad5*				*i2*
	*nad6*	*i1*^*rt*^			
	*atp6*				*i1*^*rt*^

Group I introns in the mt genomes of *C*. *austroafricana*, *C*. *cubensis*, *C*. *deuterocubensis* and *C*. *parasitica* contained 27, 6, 10 and 15 ORFs that encoded HEGs of the LAGLIDADG family, while 13, 6, 9 and 11 introns contained ORFs encoding for GIY-YIG type HEGs, respectively. Based on expression analysis of *C*. *austroafricana* RNA-Seq data (Mangwanda *et al*. submitted), these ORFs seem to be expressed (data not shown). 15, 6, 9 and 17 of these intron encoded ORFs in *C*. *austroafricana*, *C*. *cubensis*, *C*. *deuterocubensis* and *C*. *parasitica* respectively were in frame and did not have typical start codons. In the mt genomes of *C*. *austroafricana*, *C*. *deuterocubensis* and *C*. *parasitica*, 8, 2 and 4 introns respectively had two ORFs that encoded either LAGLIDADG or GIY-YIG type HEGs, or both ORFs encoded the same type of HEG (Table 2). Comparative analysis of these intron encoded HEGs in each species revealed sequence identities below 45% among themselves (data not shown). However, results from comparative analysis of these HEGs between the four species revealed homologous sequences with identities of up to 80% (S4 Table). Comparative BLAST analysis of the amino acid sequences of these HEGs against sequences deposited in the NCBI GenBank database revealed sequence identities of up to over 70% and alignment coverage of at least 50% (S5 Table).

Group II introns were not identified in the mt genomes of *C*. *cubensis* and *C*. *deuterocubensis*, however, three and five group II introns were identified in the mt genomes of *C*. *austroafricana* and *C*. *parasitica* (Tables 1 and 2). Of these group II introns only one intron in *C*. *austroafricana* mt genome had the typical reverse transcriptase encoding ORF commonly identified in this group of introns [37, 57]. In contrast four out of five group II introns in *C*. *parasitica* harboured an ORF encoding a reverse transcriptase protein (Table 2).

### Intergenic and unique ORFs

Varying numbers of intergenic ORFs were predicted in the four fungal mt genomes: 21, 7, 17 and 14 free standing ORFs were detected from *C*. *austroafricana*, *C*. *cubensis*, *C*. *deuterocubensis* and *C*. *parasitica*, respectively. In the *C*. *austroafricana* mt genome, 13 of the 21 ORFs were identified as HEGs by means of their conserved GIY-YIG and LAGLIDADG domains. Two out of the seven intergenic ORFs predicted in *C*. *cubensis* were GIY-YIG and LAGLIDADG HEGs. Of the 17 and 14 intergenic ORFs in *C*. *deuterocubensis* and *C*. *parasitica*, respectively, three ORFs were GIY-YIG type HEGs in both mt genomes, while ORFs encoding LAGLIDADG type HEGs were four and five, respectively. BLAST analysis revealed that most of these ORFs could have been acquired independently due to lack of identities between the mt genomes. However, some intergenic ORFs encoding HEGs were shared. For example, orf368 of *C*. *austroafricana* encoding a LAGLIDADG type HEG shared 99% and 94% sequence identity with orf383 of *C*. *deuterocubensis* and orf427 of *C*. *parasitica*, respectively (S4 Table). In each mt genome, intergenic HEG sequences were compared with intron encoded HEGs to determine if there was any signature of recent movement within the genome. Results from the BLAST analysis revealed sequence identities below 50%. However, BLAST analysis of predicted proteins sequences from HEGs against mt genomes deposited in GenBank revealed hits of up to over 90% sequence identities (S5 Table).

In the mt genomes of *C*. *austroafricana*, *C*. *cubensis*, *C*. *deuterocubensis* and *C*. *parasitica*, 8, 4, 12 and 12 unique ORFs were identified, respectively. These ORFs had translation start sites similar to other mt genes and translated to amino acid sequences longer than 100 residues. Comparative amino acid sequence analysis of these ORFs identified one ORF that was shared across all *Chrysoporthe* species, but was not found in the *C*. *parasitica* mt genome. This ORF (orf199) was located immediately after the *cob* gene in all three mt genomes and was 600 bp in size. Sequence alignment of these three ORFs revealed sequence identities of up to 99%. BLASTP searches of this unique ORF in the NCBI protein database did not reveal sequence alignments with high sequence coverage or identity. Searches for functional domains in Pfam and InterPro protein databases were also unsuccessful for this ORFs.

### Comparative analysis of mt genome size variation

Size polymorphism is a prominent feature of fungal mt genomes. Several factors have been associated with size variations including number and size invading introns and expanding intergenic regions. The mt genomes analysed in this study exhibited great size variation with *C*. *austroafricana* being the largest at 190,834 bp and *C*. *cubensis* the smallest at 89,084 bp. The mt genomes with more introns for example 35 and 32 in *C*. *austroafricana* and *C*. *parasitica* respectively had larger mt genomes compared to *C*. *cubensis* and *C*. *deuterocubensis* which had far fewer 14 and 19 (Table 1). In *C*. *austroafricana*, eleven of these were located in the *cox1* gene, contributing to its large size (21,506 bp). Similarly, the *C*. *cubensis*, *C*. *deuterocubensis* and *C*. *parasitica cox1* gene had 6, 6 and 8 introns, respectively, and this was the largest gene in all of these mt genomes (Table 3). The effect of introns on the gene sizes is evident from the mt genomes analysed (Table 1 and Table 3). Among the *Chrysoporthe* species and *C*. *parasitica* mt genomes, the longest intron (3,477 bp) was located in the *C*. *austroafricana* mt genome (intron 2 of the *nad2* gene), while the shortest intron was 492 bp located in the *cox3* gene of *C*. *parasitica* mt genome.

**Table 3:** Comparison of gene size and number of introns. Comparisons were performed for the 14 mitochondrial genes of *Chrysoporthe austroafricana*, *C*. *cubensis*, *C*. *deuterocubensis* and *Cryphonectria parasitica* involved in oxidative phosphorylation and electron transport. Refer to Fig 1 and S4 Fig for physical maps of these genomes.

	*C*. *austroafricana*			*C*. *cubensis*			*C*. *deuterocubensis*			*C*. *parasitica*		
Gene	Size•	Introns	CDS	Size•	Introns	CDS	Size•	Introns	CDS	Size•	Introns	CDS
*** atp6***	7,299	3	795	2,657	1	795	795	-	795	8602	3	795
*** atp8***	165	-	165	165	-	165	165	-	165	168	-	168
*** atp9***	225	-	225	225	-	225	225	-	225	252	-	252*
*** cox1***	21,506	11	1,695	9,950	6	1,695	9,946	6	1,695	17,582	8	1,695
*** cox2***	4,881	3	753	2,319	1	753	4,867	2	753	8,574	4	753
*** cox3***	3,113	1	810	810	-	810	810	-	810	6,723	5	810
*** cytb***	9,310	6	1,176	5,271	3	1,176	8,412	4	1,176	8,326	3	1,176
*** nad1***	5,656	3	1,182	1,182	-	1,182	6,815	3	1,182	4,738	1	1,161*
*** nad2***	7,743	2	1,728	5,401	2	1,728	4,228	1	1,728	12,488	6	1,728
*** nad3***	429	-	429	429	-	429	429	-	429	348	-	348*
*** nad4***	4,948	1	1,479	1,479	-	1,479	2,814	1	1,479	2,049	-	2,049^**†**^
*** nad4L***	4,945	-	270	270	-	270	270	-	270	270	-	270
*** nad5***	9,984	3	2,034*	3,107	1	2,103*	4,092	1	2,091*	6,462	2	1,971*
*** nad6***	5,684	2	681	681	-	681	2,867	1	699	666	-	666

**•** Gene sizes include intron sequences.

* Differences in CDS are due to the presence or absence of additional codons at the 3’ borders of genes.

**†** The *C*. *parasitica nad4* gene seems to be fused to a homing endonuclease gene (HEG). Only one ORF could be predicted which included both genes.

The total size of introns, intergenic regions and coding sequences were calculated. Introns constitute 72,468 bp (37.97%), 20,458 bp (22.96%), 33,238 bp (26.72%) and 63,373 bp (39.88%) in *C*. *austroafricana*, *C*. *cubensis*, *C*. *deuterocubensis* and *C*. *parasitica* respectively (Table 4). The percentage of intergenic sequences (including all OXPHOS genes, intronic ORFs and intergenic ORFs) was 48,854 bp (25.60%), 38,350 bp (43.05%), 43,340 bp (34.84%) and 41,930 bp (26.39%) in *C*. *austroafricana*, *C*. *cubensis*, *C*. *deuterocubensis* and *C*. *parasitica* respectively. Coding sequences constituted 49.17% (93,848 bp), 38.39% (34,202), 45.45% (56,550) and 48.30%) (76,752). The length of sequences in the region covering the ribosomal RNAs was largest in *C*. *austroafricana* and smallest in *C*. *cubensis* (Table 4). To determine factors majorly contributing to size polymorphisms in the four mt genomes, the proportion of coding sequences, intergenic sequences and introns was compared using the method described in [58]. From this analysis, introns and coding sequences were the primary source of genome size variation (Table 5). This result was consistent with BLAST comparisons in Fig 2 which showed more variation in intron regions. The comparison between *C*. *austroafricana* and *C*. *parasitica* revealed that intergenic sequences contributed more compared to comparisons between *C*. *austroafricana* and *C*. *cubensis* or *C*. *austroafricana* and *C*. *deuterocubensis*. In the *Chrysoporthe* mt genomes, the size variation of intergenic sequences ranged between 8% and 14%.

**Table 4:** Total sizes of genes, coding sequences, and intergenic regions of *C*. *austroafricana*, *C*. *cubensis*, *C*. *deuterocubensis* and *C*. *parasitica* mt genomes. The size of the intergenic region was calculated by subtracting the sum total of the 14 OXPHOS genes, *tRNAs*, *rRNAs* and *rnpb* from the mt genome size.

Species	mt genome size (bp)	OXPHOS genes (including introns)	CS	Introns	Intergenic region	rRNA genes (without introns)+*rnpb*	tRNA genes
*C*. *austroafricana*	190,834	85,888 (44.34%)	93,848 (49.17%)	72,468 (37.97%)	48,854 (25.60%)	36,228 (18.98%)	2,083 (1.09%)
*C*. *cubensis*	89,084	33,946 (38.11%)	34,202 (38.39%)	20,458 (22.96%)	38,350 (43.05%)	9,990 (11.21%)	2,010 (2.26%)
*C*. *deuterocubensis*	124,412	46,735 (37.56%)	56,550 (45.45%)	33,238 (26.72%)	43,340 (34.84%)	21,957 (17.65%)	2,018 (1.62%)
*C*. *parasitica*	158,902	77,248 (48.61%)	76,752 (48.30%)	63,373 (39.88%)	41,930 (26.39%)	26,345 (16.58%)	1,937 (1.22%)

**Table 5:** Comparison of factors contributing to genome size polymorphism. Pairwise comparisons were calculated for introns, intergenic regions (IR) and coding sequences (CS).

Species	*C*. *austroafricana* (CA)			*C*. *cubensis* (CC)			*C*. *deuterocubensis* (CD)			*C*. *parasitica* (CP)		
	Introns	IR	CS	Introns	IR	CS	Introns	IR	CS	Introns	IR	CS
**CA**				51%	10%	58%	59%	8%	56%	24%	22%	53%
**CC**	51%	10%	58%				36%	14%	63%	61%	5%	60%
**CD**	59%	8%	56%	36%	14%	63%				87%	4%	58%
**CP**	24%	22%	53%	61%	5%	60%	87%	4%	58%			

## Discussion

In this study the mt genome sequences of *C*. *austroafricana*, *C*. *cubensis* and *C*. *deuterocubensis* were determined. This is the first report of complete mt genome sequences in the genus *Chrysoporthe*. The sizes of these genomes were within the range of other fungal mt genomes [32, 33]. The diversity of mt genome size was evident in the mt genome sequences of *C*. *austroafricana*, *C*. *cubensis*, *C*. *deuterocubensis* and *C*. *parasitica*, which were 190,834 bp, 89 084 bp, 124 412 bp, and 158 902 bp in size, respectively. Notably, compared to all mt genomes in the NCBI organelle database, the *C*. *austroafricana* mt genome is the second largest fungal mt genome to date after that of *Sclerotinia borealis* [33]. The mt genomes of *C*. *austroafricana*, *C*. *cubensis*, *C*. *deuterocubensis* and *C*. *parasitica* seem plastic, containing only 40%, 34%, 35% and 42% protein CS, respectively. Large numbers of introns contributed up to 28% of the total genome size, while the remaining portion of noncoding sequence was comprised of highly variable intergenic regions.

*Chrysoporthe austroafricana*, *C*. *cubensis and C*. *deuterocubensis* are closely related species [1, 8, 9]. However, their mt genomes showed extensive size differences. Fungal mt genome size differences are largely attributed to the number and size of introns and the lengths of intergenic sequences, which consequently lead to variations associated with mt genome diversity [37, 59, 60]. In the current study, variations in mt genome size was consistent with other studies that attributed expansions of fungal mt genomes to intron sequences [25, 33, 35, 42, 61, 62]. The extent to which the intron sizes and numbers influence mt genome size variation is evident in the *cox1* gene of *C*. *austroafricana*, which was the largest reservoir of 11 introns in all four mt genomes. Comparing the *cox1* gene of *C*. *austroafricana* to those of *C*. *cubensis*, *C*. *deuterocubensis* and *C*. *parasitica*, the *C*. *austroafricana cox1* gene was more than double in size due to additional introns. This observation was not limited to *cox1* genes, but to all other mt genes harbouring these introns. Long stretches of intergenic sequences also seemed to influence the mt genome sizes of these species. In other fungi, these regions harbour ORFs that have been associated with genome size variations [33].

The mt genome sequences of *C*. *austroafricana*, *C*. *cubensis*, *C*. *deuterocubensis* and *C*. *parasitica* have highly similar gene content. These genomes encode fourteen genes associated with oxidative phosphorylation and electron transport in fungi. Also, genes encoding the *rns*, *rnl*, and tRNA genes, were contained in these mt genome sequences. This was consistent with previously described mt genome sequences [20, 25]. The mt genomes investigated in the current study all had an *rnpB* gene that was located in a similar location, immediately before the small subunit ribosomal RNA. BLAST searches for this gene in GenBank did not yield many hits which could be either because it was not identified in the fungal mt genomes. ORFs identified in the *Chrysoporthe* species mt genomes analysed contained a putative rps5/maturase fusion protein similar to that of previously identified in *C*. *parasitica*. Phylogenetic analysis however showed that these rps5/maturase like ORFs grouped together with rps3/HEG fusion proteins. The *rps3* gene is commonly identified in other *Sordariomycetes* [22, 24, 42].

Gene order in the four mt genomes investigated in the current study was highly conserved, despite the considerable size differences. This was in agreement with previous observations that mt genome size variations do not affect gene order [15]. When compared to mt genomes of other *Sordariomycetes*, a comparable level of synteny was observed, especially with regard to genes known to occur in pairs, such as *nad4L-nad5*, *cox3-nad6*, *nad1-nad4*, *nad2-nad3*, *atp8-atp6* and *cob-cox1* [15, 22]. A high level of gene order conservation was also observed for tRNA genes which were clustered the same as in other *Sordariomycetes* [12, 15, 42]. The organisation of tRNA genes was highly conserved, with differences emerging only due to intervening ORFs. The higher number of tRNAs in *C*. *austroafricana* mt genome was due to duplicated tRNAs that were absent in the mt genomes of *C*. *cubensis*, *C*. *deuterocubensis* and *C*. *parasitica*.

Results of this study revealed duplications of some mt genome regions. One such duplication was a 2,718 bp ORF with sequences homologous to the *nad2* gene in the mt genomes of *C*. *austroafricana*, *C*. *cubensis* and *C*. *deuterocubensis*. This duplication could not be identified in the *C*. *parasitica* mt genome, suggesting that it could have occured after the divergence of *Chrysoporthe* and *Cryphonectria*. The high sequence identity (between 97–99%) of this duplication in all three genomes of *C*. *austroafricana*, *C*. *cubensis* and *C*. *deuterocubensis*, combined with the high level of synteny around the duplicated gene region, suggests a single duplication event in the most recent common ancestor, which was then transmitted vertically when these species diverged. Notably, the sequences flanking the 5’ region of this ORF included a duplicate *tRNA-M* gene, which also flanks the 5’ region of the *nad2* gene. The occurrence of these duplications in regions around tRNAs is in agreement with studies that have associated tRNA genes with editing, excision and integration capabilities in mt genomes and are also linked to horizontal gene transfer events [63].

An ORF with partially duplicated sequences of *atp8* gene was predicted in the mt genome of *C*. *deuterocubensis* and *C*. *parasitica* between *cox2* and *atp9*. The absence of this duplication in *C*. *cubensis* and *C*. *austroafricana* suggests independent evolutionary processes involving either gain or loss after the divergence of these species. The ORFs homologous to *nad3* in *C*. *austroafricana* and *C*. *parasitica* but absent in *C*. *cubensis* and *C*. *deuterocubensis* could be due to subsequent loss after these species diverged. The presence of these duplications is consistent with what has been reported for *Sclerotinia borealis* [33] where these duplications have been associated with genome expansion and in *Fusarium* species [24] contributing to mt genome diversity. The presence of duplicated genes in these mt genomes is evidence of independent evolutionary events that may have happened after the divergence of these species. No functional or phenotypic adaptations have thus far been suggested for such duplications.

Fungal mt introns show extensive diversity even among species in the same genus [19, 24, 25]. Group I introns in the four *Cryphonectriaceae* mt genomes showed known diversity of mitochondrial introns [31, 37, 39, 62]. A high level of diversity was also observed in the few group II introns identified in the mt genomes of *C*. *austroafricana* and *C*. *parasitica*. Intron content showed remarkable diversity especially within the *cox1*, *cob*, *nad1*, *nad2* and *nad5* genes. Differences in intron content can occur in species and even subspecies groups [24, 25, 42, 61, 64, 65]. Thus, the diverse range of introns and intron content in the mt genomes of *Cryphonectriaceae* seems to be a common feature with potential influence in mt genome size variations.

In fungal mt genomes, intron acquisition can occur via both vertical and horizontal transmission, and appears to occur at homologous gene positions [39, 66, 67]. A high level of sequence similarity was observed between the group I introns of the three *Chrysoporthe* mt genomes, some of which encoded similar HEGs and had similar insertion points. Introns that lacked sequence similarities could be indicative of independent evolutionary histories involving multiple acquisitions [33, 36]. The few group II introns identified in these mt genomes did not share sequence similarities, but similar sequences could be identified from other fungal species. This suggests that group II introns were acquired by horizontal transfer. The few intron sequences from the *Chrysoporthe* species mt genomes homologous to those of the *C*. *parasitica* mt genome was consistent with the notion that introns are either fixed or lost after divergence events [24, 68].

Fungal mt introns contain ORFs that encode HEGs of the LAGLIDADG or GIY-YIG families [38, 39, 69]. In frame ORFs encoding HEGs, did not have start codons a feature that is common in fungal mt genomes. These ORFs are also found free standing in genomic regions other than those related to OXPHOS [33]. Consistent with other studies [24, 61], results of this study revealed widespread diversity of these genes in *Cryphonectriaceae* mt genomes. This, coupled with the low sequence identities (< 45%) of HEGs within each mt genome seems to rule out intragenomic proliferation, indicating that these genes could have diverse origins. Similarly, low sequence identities were observed between intron encoded and free standing HEGs within and between these mt genomes. Therefore, acquisition of these genes seems to have independent evolutionary origins which was then followed by the accumulation of mutations [67, 70].

In other fungal mt genomes, unique ORFs (uORFs) have been identified and are implicated in genome size expansion [24, 42]. One uORF was shared among the three mt genomes of *Chrysoporthe* spp. This uORF (orf199) was 600 bp long in all three mt genomes, and was located immediately after the *cob* gene. Comparative analysis using both amino acid and nucleotide sequences could not reveal significant BLAST matches, thus the origin and function of this uORF could not be identified. However, this uORF seems to have been transmitted vertically among the *Chrysoporthe* spp. from the most recent common ancestor and could potentially be used as a marker for this lineage.

Analysis of factors hugely contributing to size polymorphism in the mt genomes of *C*. *austroafricana*, *C*. *cubensis*, *C*. *deuterocubensis* and *C*. *parasitica* revealed introns and coding sequences as the primary source of genome size variation. Coding sequences included sequences from the 14 OXPHOS genes, intronic ORFs and free standing ORFs. Intergenic sequences seemed to contribute more in the size variation between *C*. *austroafricana* and *C*. *parasitica*. Also, introns did not seem to contribute much between these two mt genomes which could be attributed to the high number of introns whose size difference was the least compared to *C*. *cubensis* and *C*. *deuterocubensis*. Generally, the size of the mt genomes seemed to drastically increase with increasing number of introns and coding sequences.

## Conclusions

In this study we were able to determine the mt genome sequences of *C*. *austroafricana*, *C*. *cubensis*, and *C*. *deuterocubensis*. The *C*. *austroafricana* genome was the second largest fungal mt genome to date, and the large size could be attributed to the numbers and sizes of invading introns varying size of coding sequences and intergenic regions. Despite the size polymorphism, results from comparative analyses of these genomes indicated a conserved gene order and direction of transcription. From all analysed introns, only a few introns were orthologous, while the rest seem to have been acquired independently. The general lack of high sequence identity between proteins sequences of HEGs and uORFs identified in this study but high sequence identity when compared to protein sequences in NCBI GenBank suggest possible acquisition via horizontal transfer. However, further evolutionary analysis is required for these genes. These genomes present invaluable resources for future studies focusing on the evolution and population biology of *Chrysoporthe* species.

## Supporting Information

S1 Fig Midrooted phylogenetic tree of *rnpb* genes. Rnpb genes were mannualy retrieved from annotated fungal mt genomes publicly available in GenBank. Phylogenetic analysis was performed using maximum likelihood method implemented in RAxML with HKY model of selection. Branch support was calculated using 1000 bootstrap replicates. Green boxes depict atp6 gene, red; small sub-unit of ribosomal RNA (*rns*) and dark red; small sub-unit of ribosomal RNA (*rnl*).
(PDF)

S2 Fig Maximum likelihood phylogeny of *Rps3*. Phylogenetic analysis of orf540, orf551 and orf551 from *C*. *austroafricana*, *C*. *cubensis* and *C*. *deuterocubensis* which show sequence similarity to *C*. *parasitica* S5 ribosomal protein/maturase fusion protein. Sequences used in this phylogeny were retrieved from GenBank using BLAST. The LG+G model of substitution was used. Branch support for was calculated using the bootstrap method with 1000 replicates.
(PDF)

S3 Fig RPKM values for 14 OXPHOS genes. The graph shows the average expression for genes that are involved in oxidative phosphorylation and electron transport and the *rnpb* gene of *Chrysoporthe austroafricana* grown in complete and minimal media.
(PDF)

S4 Fig Physical maps of the mt genomes of *C*. *austroafricana*, *C*. *cubensis*, *C*. *deuterocubensis* and *C*. *parasitica* showing locations of all intronic and intergenic ORFs. Intronic ORFs are depicted by yellow arrowed boxes on introns of genes where found and are labelled according to the gene and intron position. Intergenic ORFs are also depicted by yellow arrowed boxes and are labelled with “IN” prefix.
(PDF)

S1 Table Accession number and species names for sequences used for *rps3* gene phylogeny. (PDF)

S2 Table Codon usage analysis. Comparison of codon usage and tRNAs for the 14 genes involved in oxidative phosphorylation and electron transport in the mitochondrial genomes of *Chrysoporthe austroafricana*, *C*. *cubensis*, *C*. *deuterocubensis* and *Cryphonectria parasitica*.
(PDF)

S3 Table BLAST analysis of all identified introns against mt genome sequences deposited in NCBI GenBank. The best hits for each query (intron) is shown. Default BLAST parameters were used. The prefix CA, CC, CD and CP is added to the intron names for clarity.
(PDF)

S4 Table BLAST analysis of identified intron encoded and free standing HEG protein sequences. Results from all possible comparisons were calculated. Only Blast hits with a threshold of over 50% alignment coverage, 50% sequence identity and E-value of 0.00005 are shown.
(PDF)

S5 Table BLAST analysis of intronic and free standing HEG protein sequences against mt genomes deposited in NCBI GenBank database. HEG type were annotated using the NCBI Conserved Domain Database (CDD).
(PDF)
