# Supplementary material for: Intron Derived Size Polymorphism in the Mitochondrial Genomes of Closely Related Chrysoporthe Species
Source: PLoS One. 2016 Jun 6;11(6):e0156104. doi: 10.1371/journal.pone.0156104 (PMC4894602; doi:10.1371/journal.pone.0156104)

S1 Fig. Midrooted phylogenetic tree of *rnpb* genes. *Rnpb* genes were mannualy retrieved from annotated ascomycete mt genomes publicly available in GenBank. Phylogenetic analysis was performed using maximum likelihood method implemented in RAxML with HKY model of selection. Branch support was calculated using 1000 bootstrap replicates. Green boxes depict *atp6* gene, red; small sub-unit of ribosomal RNA (*rns*) and dark red; *rnpb*, gold; *cob*, yellow; *cox* genes and lawn green boxes; *nad* genes. Accession numbers of mt genomes in which annotated *rnpb* genes were retrieved are NC_016187 (*Penicillium solitum*), KR952335 (*Penicillium roqueforti*), NC_016955 (*Peltigera malacea*), NC_025200 (*Sclerotinia borealis*), NC_018044 (*Saccharomyces paradoxus*), JQ435097 (*Aspergillus nidulans*).

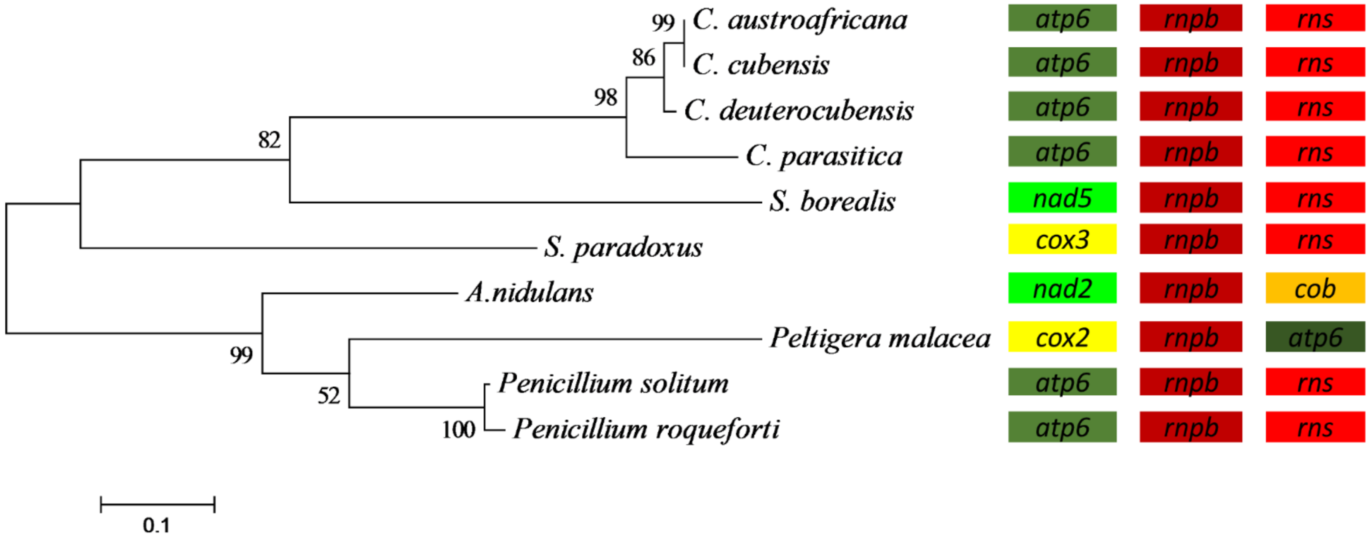

Supplement: S1 Fig — Rnpb genes were mannualy retrieved from annotated fungal mt genomes publicly available in GenBank. Phylogenetic analysis was performed using maximum likelihood method implemented in RAxML with HKY model of selection. Branch support was calculated using 1000 bootstrap replicates. Green boxes depict atp6 gene, red; small sub-unit of ribosomal RNA (rns) and dark red; small sub-unit of ribosomal RNA (rnl). (PDF) [file pone.0156104.s001.pdf]
