# Supplementary material for: Intron Derived Size Polymorphism in the Mitochondrial Genomes of Closely Related Chrysoporthe Species
Source: PLoS One. 2016 Jun 6;11(6):e0156104. doi: 10.1371/journal.pone.0156104 (PMC4894602; doi:10.1371/journal.pone.0156104)
Supplement: S2 Fig — Phylogenetic analysis of orf540, orf551 and orf551 from C. austroafricana, C. cubensis and C. deuterocubensis which show sequence similarity to C. parasitica S5 ribosomal protein/maturase fusion protein. Sequences used in this phylogeny were retrieved from GenBank using BLAST. The LG+G model of substitution was used. Branch support for was calculated using the bootstrap method with 1000 replicates. (PDF) [file pone.0156104.s002.pdf]

S2 Fig. Maximum likelihood phylogeny of *Rps3*. Phylogenetic analysis of orf540, orf551 and orf551 from *C. austroafricana*, *C. cubensis* and *C. deuterocubensis* which show sequence similarity to *C. parasitica* S5 ribosomal protein/maturase fusion protein. Sequences used in this phylogeny were retrieved from GenBank using BLAST. The LG+G model of substitution was used. Branch support for was calculated using the bootstrap method with 1000 replicates. *S3H* stands for *rps3*/HEG fusion protein. Blue stars indicate *S5* proteins sequences grouping with *rps3* proteins.

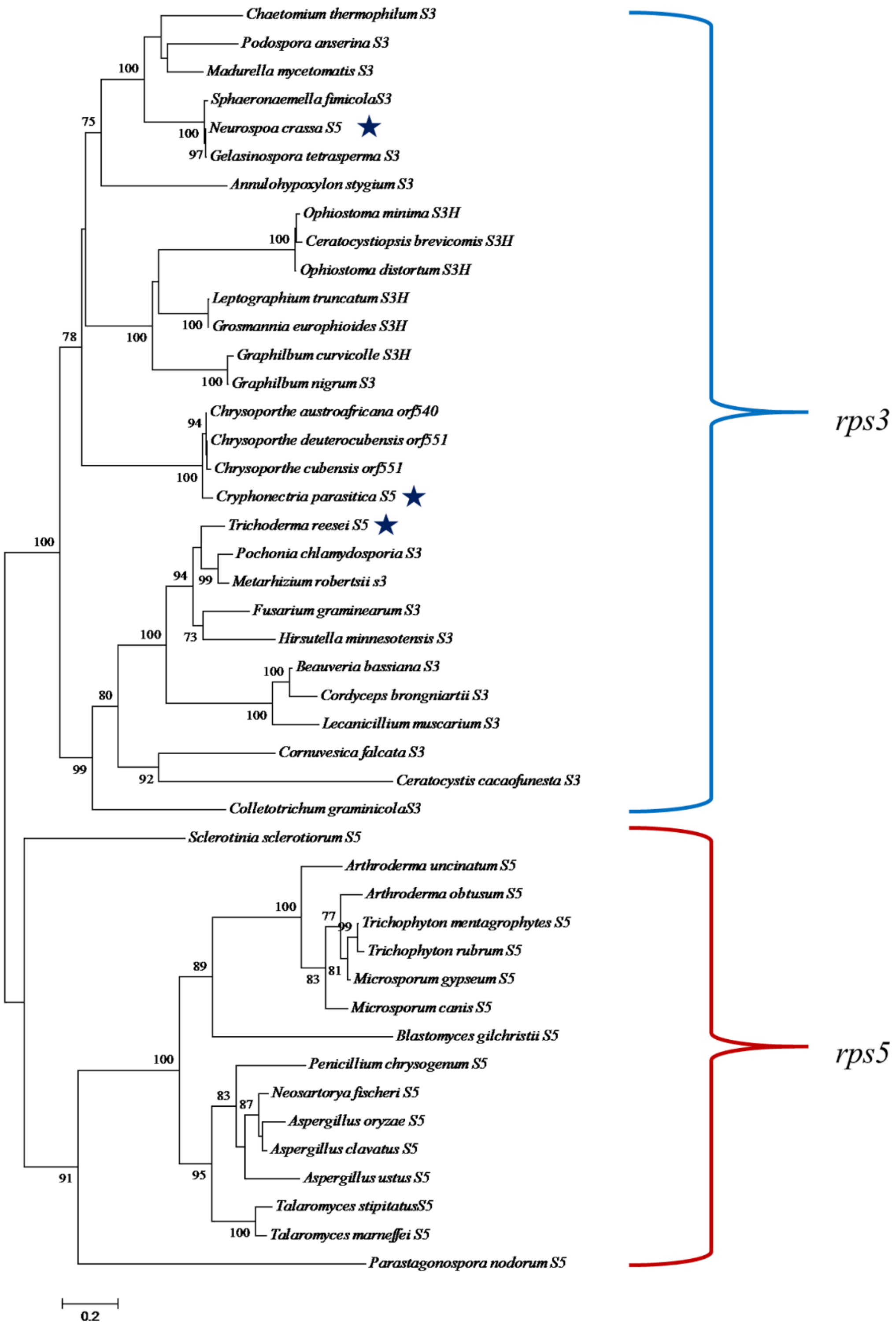
Chaetomium thermophilum S3
Podospora anserina S3
Madurella mycetomatis S3
Sphaeronaemella fimicolaS3
Neurospoa crassa S5
Gelasinospora tetrasperma S3
Annulohypoxylon stygium S3
Ophiostoma minima S3H
Ceratocystiopsis brevicomis S3H
Ophiostoma distortum S3H
Leptographium truncatum S3H
Grosmannia europhioides S3H
Graphilbum curvicolle S3H
Graphilbum nigrum S3
Chrysoporthe austroafricana orf540
Chrysoporthe deuterocubensis orf551
Chrysoporthe cubensis orf551
Cryphonectria parasitica S5
Trichoderma reesei S5
Pochonia chlamydosporia S3
Metarhizium robertsii s3
Fusarium graminearum S3
Hirsutella minnesotensis S3
Beauveria bassiana S3
Cordyceps brongniartii S3
Lecanicillium muscarium S3
Cornuvesica falcata S3
Ceratocystis cacaofunesta S3
Colletotrichum graminicolaS3
Sclerotinia sclerotiorum S5
Arthroderma uncinatum S5
Arthroderma obtusum S5
Trichophyton mentagrophytes S5
Trichophyton rubrum S5
Microsporum gypseum S5
Microsporum canis S5
Blastomyces gilchristii S5
Penicillium chrysogenum S5
Neosartorya fischeri S5
Aspergillus oryzae S5
Aspergillus clavatus S5
Aspergillus ustus S5
Talaromyces stipitatusS5
Talaromyces marneffei S5
Parastagonospora nodorum S5
rps3
rps5
0.2
