# Supplementary material for: Intron Derived Size Polymorphism in the Mitochondrial Genomes of Closely Related Chrysoporthe Species
Source: PLoS One. 2016 Jun 6;11(6):e0156104. doi: 10.1371/journal.pone.0156104 (PMC4894602; doi:10.1371/journal.pone.0156104)

S3 Fig. RPKM values for 14 genes. This graph shows the average expression for genes are involved in oxidative phosphorylation and electron transport and the *rnpb* gene of *Chrysoporthe austroafricana* grown in complete and minimal media.

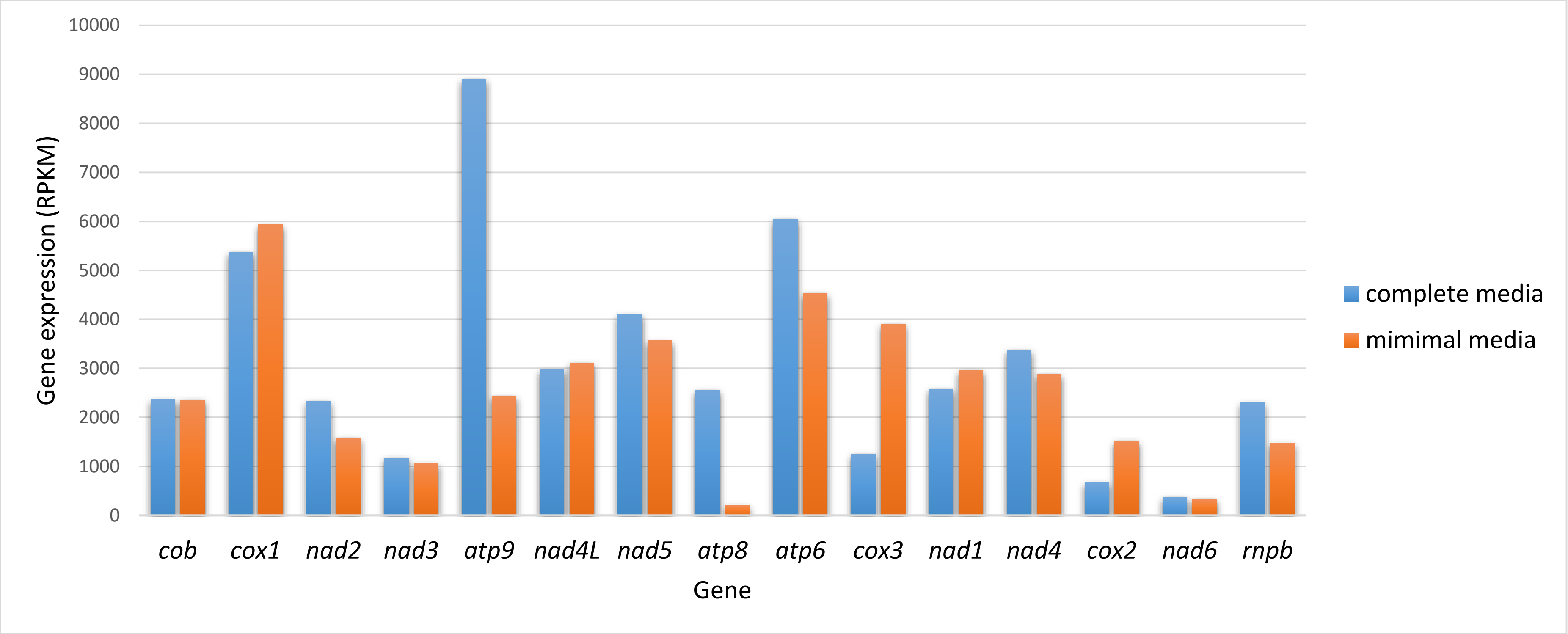

Supplement: S3 Fig — The graph shows the average expression for genes that are involved in oxidative phosphorylation and electron transport and the rnpb gene of Chrysoporthe austroafricana grown in complete and minimal media. (PDF) [file pone.0156104.s003.pdf]
