# Supplementary material for: Intron Derived Size Polymorphism in the Mitochondrial Genomes of Closely Related Chrysoporthe Species
Source: PLoS One. 2016 Jun 6;11(6):e0156104. doi: 10.1371/journal.pone.0156104 (PMC4894602; doi:10.1371/journal.pone.0156104)
Supplement: S1 Table — (PDF) [file pone.0156104.s005.pdf]

S1 Table. Accession number and species names for sequences used for *rps3* gene phylogeny.

Accession	Protein sequence description and species name
AAC24230.1	S5 ribosomal protein/maturase fusion protein *Cryphonectria parasitica*
ACV41178.1	ribosomal protein 3 *Sordaria fimicola*
YP 009126706.1	mitochondrial ribosomal protein S5 *Neurospora crassa* OR74A
ACZ97559.1	ribosomal protein 3 *Gelasinospora tetrasperma*
YP 008964947.1	ribosomal protein S3 *Annulohypoxylon styum*
YP 004769778.1	ribosomal protein S3 *Chaetomium thermophilum* var. thermophilum DSM 1495
XP 008100892.1	ribosomal protein S3 *Colletotrichum graminicola* M1.001
EXU94425.1	small subunit ribosomal protein 3 *Metarhizium robertsii*
YP 001249297.1	small ribosomal protein 3 *Fusarium graminearum*
YP 006576194.1	ribosomal protein S3 *Madurella mycetomatis*
YP 009154280.1	ribosomal protein S3 *Pseudogymnoascus pannorum*
AKK32420.1	hypothetical protein *Trichoderma harzianum*
ACV41160.1	ribosomal protein 3/homing endonuclease-like fusion protein *Ophiostoma distortum*
YP 009160634.1	putative ribosomal protein S3 *Hirsutella minnesotensis*
YP 009229331.1	ribosomal protein S3 *Verticillium nonalfalfae*
YP 009003975.1	ribosomal protein S3 *Colletotrichum lindemuthianum*
AAA31966.2	ribosomal protein S5 putative *Neurospora crassa*
ACV41151.1	ribosomal protein 3/homing endonuclease-like fusion protein *Grosmannia europhioides*
ACV41164.1	ribosomal protein 3/homing endonuclease-like fusion protein *Leptographium truncatum*
ACV41155.1	ribosomal protein 3/homing endonuclease-like fusion protein *Ceratocystiopsis brevicomis*
ACV41157.1	ribosomal protein 3/homing endonuclease-like fusion protein *Graphilbum curvicolle*
ACV41161.1	ribosomal protein 3 *Graphilbum nigrum*
ACV41175.1	ribosomal protein 3/homing endonuclease-like fusion protein *Ophiostoma minus*
YP 001876496.1	putative ribosomal protein S3 *Beauveria bassiana*
YP 004733050.1	ribosomal protein S3 *Phialocephala subalpina*
ACZ97563.1	ribosomal protein 3 *Sarcotrochila macrospora*
NP 570148.1	ribosomal protein S5 *Trichoderma reesei*
YP 008758054.1	ribosomal protein S3 *Beauveria pseudobassiana*
AKM22805.1	hypothetical protein *Cordyceps militaris*
YP 008815537.1	ribosomal protein S3 *Pochonia chlamydosporia*
YP 009072318.1	ribosomal protein S3 *Sclerotinia borealis*
YP 009122450.1	ribosomal protein S3 *Sarocladium implicatum*
XP 003009802.1	ribosomal protein S3 *Verticillium alfalfae* VaMs.102
YP 007507039.1	ribosomal protein 3 *Ceratocystis cacaofunesta*
YP 002213593.1	ribosomal protein S3 *Cordyceps brongniartii*
ACV41153.1	ribosomal protein 3/homing endonuclease-like fusion protein *Ophiostoma ulmi*

AAY59060.1	ribosomal protein 3/homing endonuclease-like protein fusion *Ophiostoma novo-ulmi subsp. americana*
AFD96021.1	ribosomal protein S5 *Asperllus clavatus*
AKQ53309.1	ribosomal protein S5 *Sclerotinia sclerotiorum* 1980 UF-70
AKQ48052.1	ribosomal protein S3 *Botrytis pseudocinerea*
AFD95940.1	ribosomal protein S5 *Neosartorya fischeri*
NP 943722.1	ribosomal protein S5 *Talaromyces marneffei*
AGN74485.1	ribosomal protein S3 *Glarea lozoyensis* 74030
AFD95916.1	ribosomal protein S5 *Talaromyces stipitatus*
AKP95616.1	ribosomal protein S3 *Penicillium nordicum*
AFD96000.1	ribosomal protein S5 *Asperllus oryzae*
EIC07753.1	ribosomal protein S3 *Microbacterium laevaniformans* OR221
YP 008965401.1	ribosomal protein S3 *Rhynchosporium orthosporum*
NP 775396.1	putative ribosomal protein S3 *Lecanicillium muscarium*
YP 008965320.1	ribosomal protein S3 *Rhynchosporium agropyri*
YP 002970834.1	ribosomal protein S5 *Arthroderma uncinatum*
YP 005351166.1	ribosomal protein subunit 3 *Peltigera malacea*
YP 009107251.1	ribosomal protein S5 *Asperllus ustus*
BAA02977.1	S5 ribosomal protein *Penicillium chrysogenum*
YP 009154205.1	ribosomal protein S3 Penicillium roqueforti
YP 002970806.1	ribosomal protein S5 *Trichophyton mentagrophytes*
XP 002620075.1	ribosomal protein S5 *Blastomyces lchristii SLH14081*
YP 002970890.1	ribosomal protein S5 *Microsporum canis*
AIG55190.1	ribosomal protein S3 *Paecilomyces hepiali*
YP 002970776.1	ribosomal protein S5 *Trichophyton rubrum*
KMW03074.1	ribosomal protein S5 *Microsporum gypseum* CBS 118893
EER36345.1	ribosomal protein S5 *Histoplasma capsulatum* H143
NP 074911.1	ribosomal protein S3 *Podospora anserina*
YP 001427397.1	ribosomal protein S5-like protein *Parastagonospora nodorum* SN15
YP 002970862.1	ribosomal protein S5 *Arthroderma obtusum*
YP 005351208.1	ribosomal protein subunit 3 *Peltigera membranacea*
ACZ97561.1	ribosomal protein 3 *Cornuvesica falcata*
ACZ97566.1	ribosomal protein 3 *Sphaeronaemella fimicola*
EEH02514.1	ribosomal protein S5 *Histoplasma capsulatum* G186AR
