# Supplementary material for: Intron Derived Size Polymorphism in the Mitochondrial Genomes of Closely Related Chrysoporthe Species
Source: PLoS One. 2016 Jun 6;11(6):e0156104. doi: 10.1371/journal.pone.0156104 (PMC4894602; doi:10.1371/journal.pone.0156104)
Supplement: S2 Table — Comparison of codon usage and tRNAs for the 14 genes involved in oxidative phosphorylation and electron transport in the mitochondrial genomes of Chrysoporthe austroafricana, C. cubensis, C. deuterocubensis and Cryphonectria parasitica. (PDF) [file pone.0156104.s006.pdf]

S2 Table. Codon usage analysis. Comparison of codon usage and tRNAs for the 14 genes involved in oxidative phosphorylation and electron transport in the mitochondrial genomes of *Chrysoporthe austroafricana*, *C. cubensis*, *C. deuterocubensis* and *Cryphonectria parasitica*.

**Amino acid**	**Codon**	***C. austroafricana***		***C. cubensis***		***C. deuterocubensis***		***C. parasitica***	
		**tRNA**[a]	***% usage***[b]	**tRNA**[a]	***% usage***[b]	**tRNA**[a]	***% usage***[b]	**tRNA**[a]	***% usage***[b]
**Ala**	GCG		0.57		0.47		0.53		0.58
**Ala**	GCA	+	1.93	+	1.72	+	1.89	+	1.66
**Ala**	GCT		3.22		3.26		3.31		3.39
**Ala**	GCC		0.53		0.53		0.49		0.44
**Cys**	TGT		0.76		0.64		0.56		0.51
**Cys**	TGC	+	0.19	+	0.14	+	0.11	+	0.18
**Asp**	GAT		2.44		2.30		2.16		2.30
**Asp**	GAC	+	0.55	+	0.35	+	0.27	+	0.35
**Glu**	GAG		0.96		0.68		0.73		0.73
**Glu**	GAA	+	2.12	+	2.09	+	1.93	+	2.06
**Phe**	TTT		5.84		6.09		6.11		5.96
**Phe**	TTC	+	2.25	+	2.50	+	2.51	+	2.24
**Gly**	GGG		1.04		0.98		0.87		0.73
**Gly**	GGA	++	2.07	+	2.09	+	2.20	+	1.82
**Gly**	GGT		3.57		3.85		3.80		4.45
**Gly**	GGC		0.14		0.12		0.11		0.27
**His**	CAT		1.19		1.23		1.16		1.20
**His**	CAC	+	0.60	+	0.64	+	0.64	+	0.60
**Ile**	ATA		5.06		5.27		5.53	+	5.05
**Ile**	ATT		4.26		4.30		4.36		4.92
**Ile**	ATC	+	0.92	+	1.15	+	1.00	+	0.97
**Lys**	AAG		0.57		0.45		0.20		0.40
**Lys**	AAA	+	3.17	+	2.34	++	2.07	+	2.39
**Leu**	TTG		1.33		1.23		1.22		1.35
**Leu**	TTA	+	9.36	+	10.18	+	10.42	+	9.99

**Leu**	CTG		0.23		0.25		0.20		0.22
**Leu**	CTA	+	1.06	+	0.96	+	1.11	+	1.24
**Leu**	CTT		1.75		1.62		1.73		1.99
**Leu**	CTC		0.23		0.18		0.16		0.16
**Met**	ATG	++++	2.23	++++	2.46	++++	2.60	+++	2.37
**Asn**	AAT		4.07		4.00		3.96		4.30
**Asn**	AAC	+	0.92	+	0.94	+	0.89	+	0.60
**Pro**	CCG		0.11		0.08		0.20		0.18
**Pro**	CCA		1.08	+	1.05		0.98	+	0.75
**Pro**	CCT	+	2.03		2.19	+	2.04		2.17
**Pro**	CCC		0.16		0.20		0.22		0.20
**Gln**	CAG		0.27		0.18		0.24		0.18
**Gln**	CAA	+	1.84	+	1.74	+	1.71	+	1.68
**Arg**	AGG		0.18		0.14		0.09		0.09
**Arg**	AGA	++	1.65	++	1.68	+	1.64	+	1.37
**Arg**	CGG		0.12		0.08		0.09		0.09
**Arg**	CGA		0.11		0.14		0.16		0.16
**Arg**	CGT	+	0.41	+	0.37	+	0.24	+	0.33
**Arg**	CGC		0.04		0.04		0.00		0.02
**Ser**	AGT		3.27		3.36		3.49		3.46
**Ser**	AGC	+	0.46	+	0.53	+	0.51	+	0.49
**Ser**	TCG		0.16		0.20		0.18		0.31
**Ser**	TCA	+	2.02	+	2.07	+	1.98	+	1.64
**Ser**	TCT		2.60		2.38		2.38		2.64
**Ser**	TCC		0.39		0.25		0.27		0.16
**Thr**	ACG		0.21		0.27		0.27		0.22
**Thr**	ACA	+	2.02	+	1.84	+	1.91	+	2.15
**Thr**	ACT		2.53		2.68		2.73		2.48
**Thr**	ACC		0.28		0.29		0.33		0.22
**Val**	GTG		0.60		0.66		0.64		0.84
**Val**	GTA	+	2.94	+	2.77	+	2.91	+	2.66

**Val**	GTT		3.04		3.24		3.22		3.23
**Val**	GTC		0.18		0.16		0.18		0.42
**Trp**	TGG		0.09		0.08		0.07		0.07
**Trp**	TGA	+	1.20	+	1.29	+	1.31	+	1.28
**Tyr**	TAT		3.54		3.55		3.62		3.59
**Tyr**	TAC	+	1.13	+	1.19	+	1.22	+	1.22
**End**	TAG		0.07		0.04		0.07		0.09
**End**	TAA		0.18		0.25		0.24		0.22

a tRNA gene for specified amino acid,

b Percentage codon usage calculated from the total number of codons of standard mt genes.

+ Presence and number of *tRNA* genes. The number of plus signs corresponds to the frequency of the respective *tRNA* gene.
