# Supplementary material for: Intron Derived Size Polymorphism in the Mitochondrial Genomes of Closely Related Chrysoporthe Species
Source: PLoS One. 2016 Jun 6;11(6):e0156104. doi: 10.1371/journal.pone.0156104 (PMC4894602; doi:10.1371/journal.pone.0156104)
Supplement: S3 Table — The best hits for each query (intron) is shown. Default BLAST parameters were used. The prefix CA, CC, CD and CP is added to the intron names for clarity. (PDF) [file pone.0156104.s007.pdf]

S3 Table. BLAST analysis of all identified introns against mt genome sequences deposited in NCBI GenBank. The best hits for each query (intron) is shown**.** Default blast parameters were used. The prefix CA, CC, CD and CP is added to the intron names for clarity.

**Intron (Query)**	**Top hit Accession**	**Description**	**E-value**	**% Identity**	**Query length**	**Alignment length**
CA_COBI1	AB027350	*Ophiocordyceps sobolifera* mitochondrial gene for small subunit ribosomal RNA	7.083E-31	72,05	1478	293
CA_COBI2	HM149254	*Monilinia fructigena isolate* ES41 cytochrome b (cytb) gene, complete cds	0	90,52	1289	486
CA_COBI3	FO906979	*Leptosphaeria maculans* brassicae wa74_scaffold00107 complete sequence	1.199E-21	93,51	1114	190
CA_COBI4	JQ015302	*Madurella mycetomatis* mitochondrion, complete genome	1.552E-140	86,81	1374	598
CA_COBI5	KC683708	*Neurospora crassa* OR74A mitochondrion, complete genome	0	84,34	1243	1169
CA_COBI6	JN204426	*Ustilaginoidea virens* isolate UV-8a Cytb protein (Cytb) gene, complete cds; mitochondrial	0	86,49	1636	836
CA_COX1I1	KT283062	*Sclerotinia sclerotiorum* 1980 UF-70 mitochondrion, complete genome	0	91,18	2924	903
CA_COX1I2	JX985789	*Tricholoma matsutake* mitochondrion, complete genome	2.058E-17	96,30	1160	142
CA_COX1I3	JN007486	*Chaetomium thermophilum* var. thermophilum strain DSM 1495 mitochondrion, complete genome	3.464E-141	89,47	2643	685
CA_COX1I4	JQ354999	*Aspergillus clavatus* strain NRRL 1 mitochondrion, partial genome	3.239E-173	76,52	1275	882
CA_COX1I5	KP137411	*Diaporthe longicolla* mitochondrion, complete genome	0	88,37	2647	571
CA_COX1I6	KR109212	*Ganoderma applanatum* strain CGMCC5.249 mitochondrion, complete genome	1.061E-128	78,38	1332	911
CA_COX1I7	FO906979	*Leptosphaeria maculans* brassicae wa74_scaffold00107 complete sequence	0	84,75	1324	1327
CA_COX1I8	KF545917	*Annulohypoxylon stygium* mitochondrion, complete genome	0	91,84	2664	1026
CA_COX1I9	GU723273	*Trametes cingulata* strain ATCC 26747 mitochondrion, complete genome	0	85,89	1085	961
CA_COX1I10	JN007486	*Chaetomium thermophilum* var. thermophilum strain DSM 1495 mitochondrion, complete genome	0	90,74	1181	870

CA_COX1I11	KT283062	*Sclerotinia sclerotiorum* 1980 UF-70 mitochondrion, complete genome	0	84,65	1576	1517
CA_NAD2I1	KC993182	*Pichia kluyveri* strain CBS 7907 mitochondrion, complete genome	1.793E-41	80,49	2538	199
CA_NAD2I2	KJ434027	*Sclerotinia borealis* strain F-4128 mitochondrion, complete genome	2.176E-116	96,77	3477	666
CA_COX2I1	FO906979	*Leptosphaeria maculans* brassicae wa74_scaffold00107 complete sequence	0	90,00	1566	805
CA_COX2I2	KJ434027	*Sclerotinia borealis* strain F-4128 mitochondrion, complete genome	0	90,91	2562	1674
CA_NAD4LI 1	JN088164	*Peltigera malacea* mitochondrion, complete genome	0	82,54	4675	654
CA_NAD5I1	AF456838	*Cryphonectria parasitica* isolate Ep 155 NADH	0	89,10	2118	2086
CA_NAD5I2	FO906020	*Leptosphaeria maculans* lepidii ibcn84_scaffold00004 complete sequence	0	88,00	4828	1521
CA_NAD5I3	KR952337	*Fusarium oxysporum* strain UASWS AC1 mitochondrion, complete genome	7.667E-100	84,16	1004	383
CA_ATP6I1	HQ259115	*Moniliophthora roreri* mitochondrion, complete genome	3.975E-78	70,21	1444	729
CA_ATP6I2	KF545917	*Annulohypoxylon stygium* mitochondrion, complete genome	0	100,00	1694	1134
CA_ATP6I1	FO905844	*Leptosphaeria biglobosa* Thlaspii ibcn65_scaffold00057 complete sequence	0	100,00	3366	1030
CA_CO3I1	NW_003533854	*Leptosphaeria maculans*	0	74,58	2303	1581
CA_NAD6I1	KU050083	*Racomitrium lanuginosum* mitochondrion, complete genome	7.031E-8	95,45	2835	44
CA_NAD6I2	FO906979	*Leptosphaeria maculans*	7.987E-96	90,24	2170	646
CA_NAD1I1	KF545917	*Annulohypoxylon stygium* mitochondrion, complete genome	0	85,28	1975	1770
CA_NAD1I2	KF545917	*Annulohypoxylon* stygium mitochondrion, complete genome	0	100,00	1406	1114
CA_NAD1I3	KJ434027	*Sclerotinia borealis* strain F-4128 mitochondrion, complete genome	1.143E-26	73,95	1093	638
CA_NAD4I1	KR952336	*Penicillium nordicum* strain UASWS BFE487 mitochondrion	1.684E-176	74,29	3469	1011
**Intron (Query)**	**Top hit accession**	**Description**	**E-value**	**% identity**	**Query length**	**Alignment length**
CD_COBI1	AB027350	*Ophiocordyceps sobolifera* mitochondrial gene for small subunit ribosomal RNA	4.498E-27	77.61	1487	294

CD_COBI2	HQ259115	*Moniliophthora roreri* mitochondrion, complete genome	0	88.82	1437	686
CD_COBI3	KC683708	*Neurospora crassa* OR74A mitochondrion, complete genome	0	84.33	1243	1169
CD_COBI4	HQ908793	*Monilia yunnanensis* isolate YQG10-6c cytochrome b (cytb) gene	0	79.60	3069	1058
CD_COX1I1	JN007486	*Chaetomium thermophilum* var. thermophilum strain DSM 1495	0	89.47	1562	925
CD_COX1I2	JQ354999	*Aspergillus clavatus* strain NRRL 1 mitochondrion, partial genome	4.183E-173	76.51	1275	882
CD_COX1I3	FO906979	*Leptosphaeria maculans* brassicae wa74_scaffold00107 complete sequence	0	79.88	1324	1327
CD_COX1I4	KF545917	*Annulohypoxylon stygium* mitochondrion, complete genome	0	92.85	1334	1026
CD_COX1I5	JN007486	*Chaetomium thermophilum var. thermophilum* strain DSM 1495	0	90.74	1180	870
CD_COX1I6	KT283062	*Sclerotinia sclerotiorum* 1980 UF-70 mitochondrion, complete genome	0	84.71	1576	1517
CD_NAD2I1	KC993182	*Pichia kluyveri* strain CBS 7907 mitochondrion, complete genome	8.784E-42	80.48	2500	199
CD_COX2I1	FO906979	*Leptosphaeria maculans brassicae* wa74_scaffold00107 complete sequence	0	90.00	1574	805
CD_COX2I2	KJ434027	*Sclerotinia borealis* strain F-4128 mitochondrion, complete genome	0	87.14	2540	1240
CD_NAD5I1	FO906020	*Leptosphaeria maculans*	6.466E-147	83.56	2001	558
CD_NAD6I1	FO906020	*Leptosphaeria maculans*	7.41E-45	92.68	2168	393
CD_NAD1I1	KF545917	*Annulohypoxylon stygium* mitochondrion, complete genome	0	88.46	2034	719
CD_NAD1I2	NW_003533854	*Leptosphaeria maculans*	0	93.75	2338	1258
CD_NAD1I3	KC683708	*Neurospora crassa* OR74A mitochondrion, complete genome	0	91.83	1261	1044
CD_NAD4I1	HQ259115	*Moniliophthora roreri* mitochondrion, complete genome	1.741E-65	79.73	1335	286
**Intron (Query)**	**Top hit Accession**	**Description**	**E-value**	**% identity**	**Query length**	**Alignment length**
CC_COBI1	AB027350	*Ophiocordyceps sobolifera* mitochondrial gene for small subunit ribosomal RNA	7.08E-31	72.05	1478	293
CC_COBI12	JQ015302	*Madurella mycetomatis* mitochondrion, complete genome	1.551E-140	86.80	1374	598

CC_COBI3	KC683708	*Neurospora crassa* OR74A mitochondrion, complete genome	0	84.33	1243	1169
CC_COX1I1	JN007486	*Chaetomium thermophilum var. thermophilum* strain DSM 1495 mitochondrion, complete genome	0	89.47	1562	925
CC_COX1I2	KP137411	*Diaporthe longicolla* mitochondrion, complete genome	0	88.39	1327	951
CC_COX1I3	FO906979	*Leptosphaeria maculans* brassicae	0	84.74	1324	1327
CC_COX1I4	KF545917	*Annulohypoxylon stygium* mitochondrion, complete genome	0	91.42	1285	1026
CC_COX1I5	JN007486	*Chaetomium thermophilum var. thermophilum* strain DSM 1495 mitochondrion, complete genome	0	90.74	1181	870
CC_COX1I6	KT283062	*Sclerotinia sclerotiorum* 1980 UF-70 mitochondrion, complete genome	0	84.58	1576	1517
CC_NAD2I1	JX271275	*Agaricus bisporus var. bisporus* H97 mitochondrion, complete genome	8.616E-62	75.44	2524	781
CC_NAD2I2	XM_003342324	*Sordaria macrospora* k-hell hypothetical protein (SMAC_12650), mRNA	4.744E-160	84.23	1152	571
CC_COX2I1	FO906979	*Leptosphaeria maculans brassicae*	0	90.00	1566	805
CC_NAD5I1	KR952337	*Fusarium oxysporum* strain UASWS AC1 mitochondrion, complete genome	2.464E-100	84.15	1004	383
CC_ATP6I1	EU053989	*Phaeosphaeria nodorum* SN15 mitochondrion, complete genome	0	100	1862	1075
**Intron (Query)**	**Top hit Accession**	**Description**	**E-value**	**% identity**	**Query length**	**alignment length**
CP_COBI1	AB027350	*Ophiocordyceps sobolifera* mitochondrial gene for small subunit ribosomal RNA	3.953E-28	76.62	1590	360
CP_COBI12	KC683708	*Neurospora crassa* OR74A mitochondrion, complete genome	0	85.96	2510	749
CP_COBI3	HQ908793	*Monilia yunnanensis* isolate YQG10-6c cytochrome b (cytb) gene, complete cds; mitochondrial	0	80.84	3050	1058
CP_COX1I1	JN007486	*Chaetomium thermophilum var. thermophilum* strain DSM 1495 mitochondrion, complete genome	0	94.11	2592	1871
CP_COX1I2	FO905844	*Leptosphaeria biglobosa*	0	79.17	2403	1473
CP_COX1I3	KF673550	*Ganoderma sinense* mitochondrion, complete genome	1.998E-53	97.36	2606	570
CP_COX1I4	KF545917	*Annulohypoxylon stygium* mitochondrion, complete genome	0	83.14	1507	990
CP_COX1I5	KP137411	*Diaporthe longicolla* mitochondrion, complete genome	7.405E-173	91.37	2545	503

CP_COX1I6	FO906979	*Leptosphaeria maculans brassicae*	0	79.12	1328	1333
CP_COX1I7	JN007486	*Chaetomium thermophilum* var. thermophilum strain DSM 1495 mitochondrion, complete genome	6.942E-148	71.18	1342	1061
CP_COX1I8	XM_007720497	*Bipolaris zeicola* 26-R-13 hypothetical protein partial mRNA	4.117E-62	78.31	1531	340
CP_NAD2I1	KC832409	*Botryotinia fuckeliana* B05.10 mitochondrion, complete genome	0	83.65	1608	924
CP_NAD2I2	KF545917	*Annulohypoxylon stygium* mitochondrion, complete genome	0	96.77	1458	1258
CP_NAD2I3	JX271275	*Agaricus bisporus* var. bisporus H97 mitochondrion, complete genome	4.256E-110	80.76	2479	882
CP_NAD2I4	Z69899	*P.comata* mitochondrial cox1 gene	6.339E-32	85.93	2577	470
CP_NAD2I5	KR011238	*Fusarium graminearum* strain CBS 104.09 mitochondrion, complete genome	0	81.63	1525	1278
CP_NAD2I6	XM_003342324	*Sordaria macrospora* k-hell hypothetical protein (SMAC_12650), mRNA	1.927E-171	84.84	1113	574
CP_COX2I1	FO906979	*Leptosphaeria maculans brassicae*	4.632E-179	92.00	1541	805
CP_COX2I2	KJ434027	*Sclerotinia borealis* strain F-4128 mitochondrion, complete genome	1.52E-96	89.18	624	356
CP_COX2I3	KJ434027	*Sclerotinia borealis* strain F-4128 mitochondrion, complete genome	0	87.35	3748	1032
CP_COX2I4	KC832409	*Botryotinia fuckeliana* B05.10 mitochondrion, complete genome	0	87.93	1908	1051
CP_NAD5I1	AF456838	*Cryphonectria parasitica* isolate Ep 155 NADH-ubiquinone oxireductase subunit 4 (ND4) gene, complete cds; NADH-ubiquinone oxireductase subunit 5, ND5 intron 1 protein and ND5 intron 2 protein (ND5) gene, complete cds; ATPase 8 gene, complete cds; and ATPase 6 gene, partial cds; mitochondrial genes for mitochondrial products	0	99.90	2091	2093
CP_NAD5I2	AF456838	*Cryphonectria parasitica* isolate Ep 155 NADH-ubiquinone oxireductase subunit 4 (ND4) gene, complete cds; NADH-ubiquinone oxireductase subunit 5, ND5 intron 1 protein and ND5 intron 2 protein (ND5) gene, complete cds; ATPase 8 gene, complete cds; and ATPase 6 gene, partial cds; mitochondrial genes for mitochondrial products	0	100	2400	2400

CP_ATP6I1	AF218567	*Cryphonectria parasitica* putative maturase (maturase) gene, complete cds; mitochondrial gene for mitochondrial product	0	99.78	2735	2366
CP_ATP6I2	KF545917	*Annulohypoxylon stygium* mitochondrion, complete genome	0	78.13	1710	1135
CP_ATP6I3	FO905844	*Leptosphaeria biglobosa* Thlaspii ibcn65_scaffold00057 complete sequence	0	93.93	3362	1030
CP_COX3I1	AF456845	*Cryphonectria parasitica* isolate I-12b NADH-ubiquinone oxireductase subunit 5, ND5 group II intron protein and ND5 intron 2 protein (ND5) gene, partial cds; mitochondrial gene for mitochondrial product	1.513E-23	84.68	942	148
CP_COX3I2	KF545917	*Annulohypoxylon stygium* mitochondrion, complete genome	1.715E-149	78.64	1178	917
CP_COX3I3	JX185564	*Ceratocystis cacaofunesta* mitochondrion, complete genome	8.373E-8	88.57	492	98
CP_COX3I4	JN041209	*Fusarium solani* mitochondrion, complete genome	0	85.36	2577	1140
CP_COX3I5	KT283062	*Sclerotinia sclerotiorum* 1980 UF-70 mitochondrion, complete genome	2.106E-97	93.33	724	374
CP_NAD1I1	KJ434027	*Sclerotinia borealis* strain F-4128 mitochondrion, complete genome	0	79.72	3577	1427
