# Supplementary material for: Intron Derived Size Polymorphism in the Mitochondrial Genomes of Closely Related Chrysoporthe Species
Source: PLoS One. 2016 Jun 6;11(6):e0156104. doi: 10.1371/journal.pone.0156104 (PMC4894602; doi:10.1371/journal.pone.0156104)
Supplement: S4 Table — Results from all possible comparisons were calculated. Only Blast hits with a threshold of over 50% alignment coverage, 50% sequence identity and E-value of 0.00005 are shown. (PDF) [file pone.0156104.s008.pdf]

S4 Table. BLAST analysis of identified intron encoded and free standing HEG protein sequences. Results from all possible comparisons were calculated.

Only Blast hits with a threshold of over 50% alignment coverage, 50% sequence identity and E-value of 0.00005 are shown.

**Query (*C. cubensis* HEG)**	**% identity**	**Alignment length**	**E-value**	**Best hit (*C. austroafricana* HEGs)**	**Query (HEG) length**
cc_IN_orf203	100.00	195	4.05612e-143	ca_IN_orf195	203
cc_cob_i1_orf361	100.00	360	0	ca_cob_i1_orf361	360
cc_cob_i2_orf344	100.00	344	0	ca_cob_i4_orf344	344
cc_cob_i3_orf305	100.00	305	0	ca_cob_i5_orf305	305
cc_IN_orf199	100.00	199	2.74836e-147	ca_IN_orf199	199
cc_cox1_i1_orf310	99.24	132	6.1544e-090	ca_cox1_i3_orf132	310
cc_cox1_i3_orf434	100.00	434	0	ca_cox1_i7_orf434	434
cc_cox1_i2_orf306	100.00	173	9.8248e-126	ca_cox1_i5_orf173	306
cc_cox1_i4_orf279	100.00	279	0	ca_cox1_i8_orf279	279
cc_cox1_i5_orf269	100.00	269	0	ca_cox1_i10_orf269	269
cc_cox1_i6_orf428	99.30	428	0	ca_cox1_i11_orf428	428
cc_IN_orf316	98.41	314	0	ca_IN_orf362	316
cc_IN_orf325	99.69	325	0	ca_cox2_i1_orf325	325
cc_IN_orf198	100.00	198	2.61646e-139	ca_IN_orf198	198
cc_nad5_i1_orf302	100.00	302	0	ca_nad5_i13_orf302	302
cc_atp6_i1_orf257	99.12	228	5.04123e-170	ca_atp6_i3_orf237	257
cc_IN_orf203	99.62	264	0	ca_atp6_i1_orf452	264
cc_IN_orf203	100.00	195	4.05612e-143	ca_IN_orf195	203
cc_IN_orf245	99.59	245	2.64309e-179	ca_rnl_orf245	245
cc_rnl_orf246	99.59	246	0	ca_rnl_orf246	246
cc_rnl_orf551	99.06	533	0	ca_rnl_orf540	551
**Query (*C. deuterocubensis* HEGs)**	**% identity**	**Alignment length**	**E-value**	**Best hit (*C. austroafricana* HEGs)**	**Query (HEG) length**
cd_cob_i1_orf364	95.59	363	0	ca_cob_i1_orf361	363
cd_cob_i2_orf350	81.95	349	0	ca_cob_i4_orf344	350
cd_cob_i3_orf305	99.02	305	0	ca_cob_i5_orf305	305

cd_IN_orf199	97.49	199	5.54481e-142	ca_IN_orf199	199
cd_cox1_i1_orf310	99.24	132	5.46179e-090	ca_cox1_i3_orf132	310
cd_cox1_i2_orf369	99.73	369	0	ca_cox1_i4_orf369	369
cd_cox1_i3_orf434	97.93	434	0	ca_cox1_i7_orf434	434
cd_cox1_i4_orf294	92.52	294	0	ca_cox1_i8_orf279	294
cd_cox1_i5_orf269	99.26	269	0	ca_cox1_i10_orf269	269
cd_cox1_i6_orf428	97.66	428	0	ca_cox1_i11_orf428	428
cd_cox2_i1_orf325	97.85	325	0	ca_cox2_i1_orf325	325
cd_cox2_i2_orf459	92.25	258	6.23259e-179	ca_cox2_i2_orf259	459
cd_IN_orf459	97.82	459	0	ca_IN_orf459	459
cd_IN_orf137	97.73	44	5.30154e-025	ca_IN_orf198	137
cd_nad5_i1_orf486	90.00	70	5.59979e-038	ca_nad5_i2_orf99	486
cd_IN_orf203	99.24	264	0	ca_atp6_i1_orf452	264
cd_rns_orf333	75.62	320	5.72715e-179	ca_rns_orf326	333
cd_rns_orf171	92.13	127	2.59967e-082	ca_rns_orf373	171
cd_rns_orf249	92.13	127	2.59967e-082	ca_rns_orf373	171
cd_rns_orf297	100.00	297	0	ca_IN_orf297	297
cd_nad6_i1_orf551	97.67	473	0	ca_nad6_i2_orf473	551
cd_IN_orf383	98.36	365	0	ca_IN_orf368	383
cd_IN_orf105	62.39	109	4.3586e-040	ca_IN_orf118	105
cd_IN_orf219	63.59	206	8.44336e-091	ca_IN_orf211	219
cd_IN_orf143	61.81	144	1.33168e-042	ca_IN_orf147	143
cd_IN_orf153	80.39	153	4.1264e-089	ca_IN_orf153	153
cd_IN_orf106	72.04	93	3.58606e-044	ca_IN_orf248	106
cd_IN_orf179	93.30	179	1.58984e-117	ca_rnl_orf245	179
cd_rnl_orf197	83.42	187	4.84359e-116	ca_rnl_orf188	197
cd_rnl_orf246	98.37	246	1.70586e-179	ca_rnl_orf246	246
cd_rnl_orf459	98.69	459	0	ca_rnl_orf459	459
cd_rnl_orf551	96.81	533	0	ca_rnl_orf540	551

cd_IN_orf333	95.50	333	0	ca_IN_orf348	333
cd_IN_orf341	97.64	339	0	ca_IN_orf478	341
cd_IN_orf126	97.64	339	0	ca_IN_orf478	341
cd_nad1_i1_orf191	95.98	174	2.44444e-116	ca_nad1_i1_orf473	191
cd_nad1_i2_orf185	100.00	30	1.785e-018	ca_nad1_i2_orf152	185
cd_nad1_i2_orf100	57.78	90	2.12297e-028	ca_IN_orf369	100
cd_nad1_i3_orf122	34.78	115	2.94946e-012	ca_cox2_i1_orf325	122
cd_nad4_i1_orf432	99.02	410	0	ca_nad4_i1_orf410	432
**Query id (*C. parasitica*)**	**% Identity**	**Alignment length**	**E-value**	**Best hit (*C. austroafricana*)**	**Query (HEG) length**
cp_IN_orf160	50.00	16	0.756835	ca_nad4L_i1_orf177	160
cp_nad1_i1	59.38	128	8.814e-049	ca_nad1_i1_orf473	131
cp_rns_orf509	67.11	450	0	ca_rns_orf512	509
cp_IN_orf179	69.30	114	2.76115e-050	ca_IN_orf185	179
cp_rns_orf151	78.63	117	6.32947e-064	ca_rns_orf326	151
cp_cob_i2_orf296	79.32	295	8.09228e-174	ca_cob_i5_orf305	296
cp_cox1_i5_orf434	79.45	438	0	ca_cox1_i5_orf429	434
cp_rnl_orf194	82.22	180	7.7753e-107	ca_rnl_orf188	194
cp_rnl_orf387	82.66	346	0	ca_rnl_orf401	387
cp_cob_i1_orf356	83.71	356	0	ca_cob_i1_orf361	356
cp_cox1_i7_orf310	84.24	203	3.68425e-123	ca_cox1_i8_orf203	310
cp_cox1_i5_orf173	86.71	173	4.22695e-113	ca_cox1_i5_orf173	173
cp_IN_orf106	87.38	103	4.75303e-049	ca_IN_orf297	106
cp_rnl_orf851	87.74	530	0	ca_rnl_orf540	851
cp_nad5_i1_orf540	87.76	539	0	ca_nad5_i1_orf537	540
cp_cox2_i1_orf326	88.65	326	0	ca_cox2_i1_orf325	326
cp_atp6_i3_orf238	88.66	238	6.46194e-156	ca_atp6_i3_orf237	238
cp_rnl_orf246	88.76	249	1.29421e-160	ca_rnl_orf246	246
cp_atp6_i3_orf409	89.24	409	0	ca_atp6_i3_orf409	409

cp_cox3_i2_orf318	89.81	314	0	ca_cox3_i1_orf314	318
cp_cox1_i6_orf436	90.14	436	0	ca_cox1_i7_orf434	436
cp_IN_orf427	91.72	326	0	ca_IN_orf368	427
cp_rns_orf28	92.51	267	0	ca_rns_orf267	285
cp_IN_orf414	93.97	365	0	ca_IN_orf369	414
cp_cox2_i3_orf112	94.20	69	2.86625e-042	ca_cox2_i2_orf259	112
cp_rns_orf416	94.37	373	0	ca_rns_orf373	416
cp_atp6_i2_orf365	94.54	366	0	ca_atp6_i2_orf370	365
cp_IN_orf414	95.29	191	2.55418e-136	ca_IN_orf191	414
cp_rnl_orf459	96.17	444	0	ca_rnl_orf455	459
**Query (*C. cubensis*)**	**% Identity**	**Alignment length**	**E-value**	**Best hit (*C. deuterocubensis*)**	**Query (HEG) length**
cc_IN_orf203	95.07	203	2.3964e-142	cd_IN_orf203	203
cc_cob_i1_orf361	95.59	363	0	cd_cob_i1_orf364	360
cc_cob_i2_orf344	81.95	349	0	cd_cob_i2_orf350	344
cc_cob_i3_orf305	99.02	305	0	cd_cob_i3_orf305	305
cc_IN_orf199	97.49	199	3.13106e-142	cd_IN_orf199	199
cc_IN_orf103	89.74	78	4.02419e-047	cd_IN_orf101	103
cc_cox1_i1_orf310	99.35	310	0	cd_cox1_i1_orf310	310
cc_cox1_i3_orf434	97.93	434	0	cd_cox1_i3_orf434	434
cc_cox1_i4_orf279	92.52	294	0	cd_cox1_i4_orf294	279
cc_cox1_i5_orf269	99.26	269	0	cd_cox1_i5_orf269	269
cc_cox1_i6_orf428	97.43	428	0	cd_cox1_i6_orf428	428
cc_IN_orf325	98.15	325	0	cd_cox2_i1_orf325	325
cc_IN_orf198	97.73	44	1.74817e-024	cd_IN_orf137	198
cc_IN_orf203	99.62	264	0	cd_IN_orf203	264
cc_IN_orf203	95.07	203	2.3964e-142	cd_IN_orf203	203
cc_IN_orf245	93.30	179	1.80015e-117	cd_IN_orf179	245
cc_rnl_orf246	98.78	246	0	cd_rnl_orf246	246

cc_rnl_orf551	97.82	550	0	cd_rnl_orf551	551
**Query (*C. cubensis*)**	**% Identity**	**Alignment length**	**E-value**	**Best hit (*C. parasitica*)**	**Query (HEG) length**
cc_cob_i3_orf305	79.32	295	6.52743e-174	cp_cob_i2_orf296	305
cc_cob_i1_orf361	84.36	358	0	cp_cob_i1_orf356	360
cc_cox1_i2_orf306	86.71	173	2.10816e-111	cp_cox1_i5_orf173	306
cc_rnl_orf551	88.15	523	0	cp_rnl_orf851	551
cc_IN_orf325	88.96	326	0	cp_cox2_i1_orf326	325
cc_rnl_orf246	89.16	249	1.05408e-161	cp_rnl_orf246	246
cc_atp6_i1_orf257	89.47	228	5.83895e-152	cp_atp6_i3_orf238	257
cc_cox1_i3_orf434	90.14	436	0	cp_cox1_i6_orf436	434
**Query (*C. parasitica*)**	**% Identity**	**Alignment length**	**E-value**	**Best hit (*C. parasitica*)**	**Query (HEG) length**
cd_nad1_i1_orf191	59.38	128	3.23038e-051	cp_nad1_i1_orf131	191
cd_cob_i3_orf305	80.00	295	2.90036e-175	cp_cob_i2_orf296	305
cd_cob_i1_orf364	83.84	359	0	cp_cob_i1_orf356	363
cd_cob_i4_orf256	86.21	116	2.18369e-066	cp_cob_i3_orf120	256
cd_rns_orf297	86.54	104	1.63116e-048	cp_IN_orf106	297
cd_IN_orf383	87.82	353	0	cp_IN_orf427	383
cd_rnl_orf246	87.95	249	7.8247e-160	cp_rnl_orf246	246
cd_rnl_orf551	88.34	523	0	cp_rnl_orf851	551
cd_cox2_i1_orf325	88.65	326	0	cp_cox2_i1_orf326	325
cd_rnl_orf352	89.07	247	2.85466e-163	cp_IN_orf247	352
cd_cox1_i3_orf434	90.83	436	0	cp_cox1_i6_orf436	434
cd_cob_i4_orf202	94.00	200	6.69243e-136	cp_cob_i3_orf201	202
cd_cox2_i2_orf459	95.10	102	5.76496e-063	cp_cox2_i3_orf112	459
cd_rnl_orf197	96.11	180	1.91567e-125	cp_rnl_orf194	197
cd_rns_orf171	96.45	169	9.82424e-119	cp_rns_orf416	171
cd_rns_orf249	96.45	169	9.82424e-119	cp_rns_orf416	171
