# Supplementary material for: Intron Derived Size Polymorphism in the Mitochondrial Genomes of Closely Related Chrysoporthe Species
Source: PLoS One. 2016 Jun 6;11(6):e0156104. doi: 10.1371/journal.pone.0156104 (PMC4894602; doi:10.1371/journal.pone.0156104)
Supplement: S5 Table — HEG type were annotated using the NCBI Conserved Domain Database (CDD). (PDF) [file pone.0156104.s009.pdf]

S5 Table. BLAST analysis of intronic and free standing HEG protein sequences against mt genomes deposited in NCBI GenBank database. HEG type were annotated using the NCBI Conserved Domain Database (CDD).

**Query (intron)**	**Top Hit Accession**	**% identity**	**Alignment length**	**E-value**	**Description**	**Query length**	**HEG Type**
ca_atp6_i1_orf452	AKQ53315	48.35	424	1.63845e-115	*Sclerotinia sclerotiorum 1980 UF-70*	452	LAGLIDADG
ca _cob_i1_orf361	1703265F	49.16	299	5.98996e-90	*Podospora anserina cytochrome oxidase I intronic ORF*	360	LAGLIDADG
ca_cob_i3_orf331	CAA38805	39.07	279	5.17524e-45	*Podospora anserina*	331	LAGLIDADG
ca_cob_i4_orf344	EYE89922	79.86	288	8.15619e-155	*Aspergillus ruber CBS 135680*	344	GIY-YIG
ca_cob_i5_orf305	YP_009126715	76.51	315	5.54442e-167	*Neurospora crassa OR74A*	305	LAGLIDADG
ca_cob_i6_orf179	YP_008964970	42.11	171	1.83817e-36	*Annulohypoxylon stygium*	179	LAGLIDADG
ca_cox1_i1_orf327	AKQ53312	72.70	326	9.22196e-169	*Sclerotinia sclerotiorum 1980 UF-70*	327	LAGLIDADG
ca_cox1_i1_orf361	YP_004376358	48.28	319	1.90945e-92	*Moniliophthora roreri*	361	LAGLIDADG
ca_cox1_i2_orf240	YP_008082053	42.53	221	5.57199e-45	*Rhizoctonia solani*	240	LAGLIDADG
ca_cox1_i3_orf183	AFM36681	46.93	179	7.76828e-52	*Ophiostoma hyalothecium*	183	LAGLIDADG
ca _cox1_i3_orf132	XP_007693509	77.59	116	7.26496e-55	*Bipolaris oryzae ATCC 44560*	132	LAGLIDADG
ca_cox1_i4_orf369	CAA25371	59.67	367	9.87938e-142	*Aspergillus nidulans*	369	LAGLIDADG
ca_cox1_i5_orf429	YP_009160660	40.07	272	1.15006e-46	*Hirsutella minnesotensis*	429	GIY-YIG
ca_cox1_i5_orf173	YP_008964978	75.14	173	3.71338e-93	*Annulohypoxylon stygium*	173	LAGLIDADG
ca_cox1_i6_orf430	YP_001249330	62.56	430	1.39084e-179	*Fusarium graminearum*	430	LAGLIDADG
ca_cox1_i7_orf434	AKQ53319	76.94	425	0	*Sclerotinia sclerotiorum 1980 UF-70*	434	GIY-YIG
ca_cox1_i8_orf279	YP_008964985	80.99	263	3.95451e-147	*Annulohypoxylon stygium*	279	LAGLIDADG
ca_cox1_i8_orf203	XP_002839373	63.64	198	6.25949e-75	*Tuber melanosporum Mel28*	203	LAGLIDADG
ca_cox1_i9_orf346	YP_005088117	73.78	347	5.333e-172	*Fusarium solani*	346	LAGLIDADG
ca _cox1_i10_orf269	AFD95924	75.46	269	1.63384e-146	*Talaromyces stipitatus*	269	GIY-YIG

ca_cox1_i11_orf428	YP_008964986	77.61	451	0	*Annulohypoxylon stygium*	428	GIY-YIG
ca_IN_orf362	KIH86252	42.00	300	2.37712e-73	*Sporothrix brasiliensis 5110*	362	LAGLIDADG
ca_nad2_i2_orf714	1703265F	48.99	296	2.56279e-92	*Podospora anserina cytochrome oxidase I intronic ORF*	714	GIY-YIG
ca_IN_orf402	4Z1X_A	53.38	296	2.97005e-100	*Fusarium graminearum PH-1 Chain B, Crystal Structure Of Laglidadg Homing Endonuclease*	402	LAGLIDADG
ca_IN_orf146	YP_004778114	78.57	28	7.43104e-05	*Sporothrix schenckii*	146	LAGLIDADG
ca_IN_orf191	5E5S_A	48.19	193	1.45587e-52	*Sordaria macropora*	191	LAGLIDADG
ca_cox2_i1_orf325	YP_008854719	59.04	293	1.0262e-103	*Ganoderma sinense*	325	GIY-YIG
ca_cox2_i2_orf259	YP_009072388	80.69	259	4.31736e-151	*Sclerotinia borealis*	259	GIY-YIG
ca_cox2_i2_orf100	YP_009072388	87.63	97	5.45437e-51	*Sclerotinia borealis*	100	LAGLIDADG
ca_IN_orf459	CCO62245	29.53	359	1.3817e-39	*Rhodotorula taiwanensis RS1*	459	GIY-YIG
ca_IN_orf369	YP_001249320	52.29	218	3.7195e-70	*Fusarium graminearum*	369	LAGLIDADG
ca_IN_orf185	AGN49019	69.93	153	3.31461e-66	*Botrytis cinerea B05.10*	185	GIY-YIG
ca_IN_orf102	EMR89155	71.56	109	8.80858e-42	*Botrytis cinerea BcDW1*	102	GIY-YIG
ca_nad4L_i1_orf177	AKQ53295	72.90	155	5.30337e-67	*Sclerotinia sclerotiorum 1980 UF-70*	177	LAGLIDADG
ca_nad4L_i1_orf329	YP_009112035	48.41	314	2.70105e-89	*Fusarium gerlachii*	329	LAGLIDADG
ca_nad5_i1_orf537	NP_074944	65.56	543	0	*Podospora anserina*	537	LAGLIDADG
ca_nad5_i2_orf99	YP_008964961	77.27	44	3.25911e-15	*Annulohypoxylon stygium*	99	LAGLIDADG
ca_nad5_i2_orf354	YP_009072328	32.99	388	2.45896e-44	*Sclerotinia borealis*	354	GIY-YIG
ca_nad5_i2_orf141	AKQ53305	50.81	124	5.9065e-32	*Sclerotinia sclerotiorum 1980 UF-70*	141	GIY-YIG
ca_nad5_i13_orf302	AAW67497	69.08	304	4.68452e-141	*Fusarium oxysporum*	302	LAGLIDADG
ca_atp6_i1_orf417	AKQ53315	48.46	421	3.21309e-116	*Sclerotinia sclerotiorum 1980 UF-70*	417	LAGLIDADG
ca_atp6_i1_orf452	AKQ53315	48.35	424	1.63845e-115	*Sclerotinia sclerotiorum 1980 UF-70*	452	LAGLIDADG
ca_atp6_i2_orf370	YP_008964995	72.85	372	0	*Annulohypoxylon stygium*	370	GIY-YIG

ca_atp6_i1_orf452	YP_009158300	95.38	260	4.59532e-164	*Diaporthe longicolla*	264	LAGLIDADG
ca_atp6_i1_orf417	AKQ53315	48.46	421	3.21309e-116	*Sclerotinia sclerotiorum 1980 UF-70*	417	LAGLIDADG
ca_atp6_i1_orf452	AKQ53315	48.35	424	1.63845e-115	*Sclerotinia sclerotiorum 1980 UF-70*	452	LAGLIDADG
ca_atp6_i3_orf409	XP_003342404	65.69	376	6.10401e-159	*Sordaria macrospora k-hell*	409	GIY-YIG
ca_atp6_i3_orf237	YP_001427393	74.03	231	3.23166e-113	*Parastagonospora nodorum SN15*	237	GIY-YIG
ca_IN_orf195	EQD84148	54.17	24	2.90092	*Saccharopolyspora erythraea D*	195	LAGLIDADG
ca_rns_orf267	AAB84209	92.51	267	0	*Cryphonectria parasitica*	267	LAGLIDADG
ca_rns_orf512	AAB84210	66.76	358	3.87038e-164	*Cryphonectria parasitica*	512	LAGLIDADG
ca_rns_orf326	AAB84211	68.90	328	6.15189e-160	*Cryphonectria parasitica*	326	LAGLIDADG
ca_rns_orf373	AAB84212	94.37	373	0	*Cryphonectria parasitica*	373	LAGLIDADG
ca_IN_orf297	YP_008964996	55.69	255	1.22603e-83	*Annulohypoxylon stygium*	297	GIY-YIG
ca_cox3_i1_orf314	AGN49000	62.88	299	1.87911e-129	*Botrytis cinerea B05.10*	314	LAGLIDADG
ca_cox3_i1_orf335	YP_005088113	39.32	295	7.7595e-47	*Fusarium solani*	335	LAGLIDADG
ca_nad6_i2_orf473	YP_007507089	55.66	424	4.60049e-150	*Ceratocystis cacaofunesta*	473	LAGLIDADG
ca_IN_orf368	YP_008854715	36.08	352	3.5558e-63	*Ganoderma sinense*	368	LAGLIDADG
ca_IN_orf211	WP_025294344	40.00	35	2.74342	*Sphingomonas sanxanigenens*	211	LAGLIDADG
ca_IN_orf153	XP_009656850	25.00	84	1.69303	*Verticillium dahliae VdLs.17*	153	LAGLIDADG
ca_IN_orf248	YP_007507082	34.50	200	2.63005e-15	*Ceratocystis cacaofunesta*	248	GIY-YIG
ca_rnl_orf318	AKQ53307	49.03	206	2.96557e-58	*Sclerotinia sclerotiorum 1980 UF-70*	318	GIY-YIG
ca_rnl_orf245	AKM22523	59.17	240	7.94197e-88	*Cordyceps militaris*	245	GIY-YIG
ca_rnl_orf188	GAO18872	55.68	176	4.33611e-58	*Ustilaginoidea virens*	188	LAGLIDADG
ca_rnl_orf246	AKM22622	66.29	175	4.46394e-75	*Cordyceps militaris*	246	LAGLIDADG
ca_rnl_orf455	NP_074929	39.08	325	3.59422e-62	*Podospora anserina*	455	GIY-YIG
ca_rnl_orf401	ACL27279	44.56	294	9.50933e-78	*Ustilago maydis*	401	LAGLIDADG
ca_rnl_orf459	YP_007507038	42.48	412	4.55929e-86	*Ceratocystis cacaofunesta*	459	LAGLIDADG
ca_rnl_orf540	AAC24230	87.74	530	0	*Cryphonectria parasitica*	540	LAGLIDADG

ca_rnl_orf260	AAC24230	87.60	258	1.00703e-158	*Cryphonectria parasitica*	260	LAGLIDADG
ca_rnl_orf383	3R7P_A	43.15	336	1.30797e-71	*Chain A, The Crystal Structure Of I-ltr*	383	LAGLIDADG
ca_IN_orf348	YP_009136815	40.64	342	1.58476e-82	*Fusarium culmorum*	348	LAGLIDADG
ca_IN_orf730	CDL73348	38.06	402	8.2847e-76	*Fusarium pseudograminearum CS5834*	730	LAGLIDADG
ca_IN_orf478	YP_007507047	39.65	401	1.976e-75	*Ceratocystis cacaofunesta*	478	LAGLIDADG
ca_nad1_i1_orf473	NP_074957	61.85	464	0	*Podospora anserina*	473	GIY-YIG
ca_nad1_i2_orf152	YP_008964989	83.44	157	4.73107e-88	*Annulohypoxylon stygium*	152	LAGLIDADG
ca_nad4_i1_orf410	YP_008757687	47.50	400	1.13513e-114	*Fusarium circinatum*	410	LAGLIDADG
**Query (Intron)**	**Top Hit Accession**	**% identity**	**alignment length**	**E-value**	**Description**	**Query length**	
cc_IN_orf203	EQD84148	54.17	24	3.15127	*Saccharopolyspora erythraea*	203	LAGLIDADG
cc_cob_i1_orf361	1703265F	49.16	299	5.98996e-90	*Podospora anserina*	360	LAGLIDADG
cc_cob_i2_orf344	EYE89922	79.86	288	8.15619e-155	*Aspergillus ruber CBS 135680*	344	GIY-YIG
cc_cob_i3_orf305	YP_009126715	76.51	315	5.54442e-167	*Neurospora crassa OR74A*	305	LAGLIDADG
cc_IN_orf103	WP_005699113	26.42	53	5.78733	*Haemophilus parainfluenzae HK2019*	103	LAGLIDADG
cc_cox1_i1_orf310	XP_007693509	63.40	306	8.59778e-128	*Bipolaris oryzae ATCC 44560*	310	LAGLIDADG
cc_cox1_i3_orf434	AKQ53319	76.94	425	0	*Sclerotinia sclerotiorum 1980 UF-70*	434	GIY-YIG
cc_cox1_i2_orf306	YP_004376350	73.29	277	4.95562e-153	*Moniliophthora roreri*	306	LAGLIDADG
cc_cox1_i4_orf279	YP_008964985	80.99	263	3.95451e-147	*Annulohypoxylon stygium*	279	LAGLIDADG
cc_cox1_i5_orf269	AFD95924	75.46	269	1.63384e-146	*Talaromyces stipitatus*	269	GIY-YIG
cc_cox1_i6_orf428	YP_008964986	77.38	451	0	*Annulohypoxylon stygium*	428	GIY-YIG
cc_IN_orf316	KIH86252	38.96	249	2.93745e-52	*Sporothrix brasiliensis 5110*	316	LAGLIDADG
cc_IN_orf325	YP_008854719	59.39	293	1.05103e-104	*Ganoderma sinense*	325	GIY-YIG
cc_nad5_i1_orf302	AAW67497	69.08	304	4.68452e-141	*Fusarium oxysporum*	302	LAGLIDADG
cc_atp6_i1_orf257	YP_001427393	74.62	260	1.23618e-131	*Parastagonospora nodorum SN15*	257	GIY-YIG
cc_IN_orf203	YP_009158300	95.77	260	2.80965e-164	*Diaporthe longicolla*	264	LAGLIDADG

cc_IN_orf203	EQD84148	54.17	24	3.15127	*Saccharopolyspora erythraea*	203	LAGLIDADG
cc_IN_orf245	AKM22523	59.58	240	3.87846e-89	*Cordyceps militaris*	245	GIY-YIG
cc_rnl_orf246	AKM22622	66.29	175	1.31338e-75	*Cordyceps militaris*	246	LAGLIDADG
cc_rnl_orf551	AAC24230	88.15	523	0	*Cryphonectria parasitica*	551	LAGLIDADG
Query id	subject id	% identity	Alignment length	E-value	Description	Query length	
cd_cob_i1_orf364	1703265F	49.16	299	4.53439e-90	*Podospora anserina*	363	LAGLIDADG
cd_cob_i2_orf350	EYE89922	69.62	339	1.29869e-153	*Aspergillus ruber CBS 135680*	350	GIY-YIG
cd_cob_i3_orf305	YP_009126715	77.14	315	2.62494e-168	*Neurospora crassa OR74A*	305	LAGLIDADG
cd_cob_i4_orf256	CAB72450	69.23	221	1.02443e-98	*Podospora curvicolla*	256	GIY-YIG
cd_cob_i4_orf202	XP_007693966	38.35	206	3.7913e-30	*Bipolaris oryzae ATCC 44560*	202	GIY-YIG
cd_IN_orf199	CEJ90698	29.17	72	9.63143	*Torrubiella hemipterigena*	199	LAGLIDADG
cd_IN_orf101	WP_005699113	28.30	53	1.58061	*Haemophilus*	101	LAGLIDADG
cd_cox1_i1_orf310	XP_007693509	63.40	306	1.14145e-127	*Bipolaris oryzae ATCC 44560*	310	LAGLIDADG
cd_cox1_i2_orf369	CAA25371	59.67	367	6.01917e-141	*Aspergillus nidulans*	369	LAGLIDADG
cd_cox1_i3_orf434	AKQ53319	77.41	425	0	*Sclerotinia sclerotiorum 1980 UF-70*	434	GIY-YIG
cd_cox1_i4_orf294	YP_008964985	75.90	278	1.63718e-141	*Annulohypoxylon stygium*	294	LAGLIDADG
cd_cox1_i5_orf269	AFD95924	75.46	269	1.29904e-146	*Talaromyces stipitatus*	269	GIY-YIG
cd_cox1_i6_orf428	YP_008964986	78.05	451	0	*Annulohypoxylon stygium*	428	GIY-YIG
cd_cox2_i1_orf325	YP_008854719	60.41	293	3.59504e-106	*Ganoderma sinense*	325	GIY-YIG
Cd_cox2_i2_orf459	YP_009072388	80.44	455	0	*Sclerotinia borealis*	459	GIY-YIG
cd_IN_orf120	YP_009158299	83.67	49	1.20564e-17	*Diaporthe longicolla*	52	LAGLIDADG
cd_IN_orf130	XP_001802056	40.00	55	2.60677	*Parastagonospora nodorum SN15*	130	LAGLIDADG
cd_IN_orf459	CCO62245	29.53	359	5.31366e-40	*Rhodotorula taiwanensis RS1*	459	GIY-YIG
cd_IN_orf137	EMR89155	62.50	40	1.74333e-05	*Botrytis cinerea BcDW1*	137	LAGLIDADG
cd_nad5_i1_orf486	YP_008964961	68.57	140	3.1618e-56	*Annulohypoxylon stygium*	486	LAGLIDADG
cd_IN_orf203	YP_009158300	95.38	260	9.87819e-164	*Diaporthe longicolla*	264	LAGLIDADG
cd_rns_orf333	AAB84211	78.16	348	0	*Cryphonectria parasitica*	333	LAGLIDADG

cd_rns_orf171	AAB84212	96.45	169	1.48402e-112	*Cryphonectria parasitica*	171	LAGLIDADG
cd_rns_orf249	AAB84212	96.45	169	1.48402e-112	*Cryphonectria parasitica*	171	LAGLIDADG
cd_rns_orf297	YP_008964996	55.69	255	1.22603e-83	*Annulohypoxylon stygium*	297	GIY-YIG
cd_nad6_i1_orf551	YP_007507089	54.95	424	5.04389e-148	*Ceratocystis cacaofunesta*	551	LAGLIDADG
cd_IN_orf383	YP_009136815	39.04	333	1.41708e-63	*Fusarium culmorum*	383	LAGLIDADG
cd_IN_orf219	XP_011858455	29.46	112	9.54856	*Vollenhovia emeryi*	219	LAGLIDADG
cd_IN_orf143	WP_048008014	33.96	53	6.40209	*Bacillus firmus*	143	LAGLIDADG
cd_IN_orf106	YP_009059691	40.00	70	0.000197543	*Parasitella parasitica*	106	GIY-YIG
cd_IN_orf179	AKM22523	63.07	176	6.93825e-68	*Cordyceps militaris*	179	GIY-YIG
cd_rnl_orf352	YP_009072317	69.46	239	7.18646e-115	*Sclerotinia borealis*	352	GIY-YIG
cd_rnl_orf197	GAO18872	56.82	176	3.87308e-58	*Ustilaginoidea virens*	197	LAGLIDADG
cd_rnl_orf246	AKM22622	65.71	175	2.36427e-74	*Cordyceps militaris*	246	LAGLIDADG
cd_rnl_orf459	YP_007507038	41.99	412	2.51508e-85	*Ceratocystis cacaofunesta*	459	LAGLIDADG
cd_rnl_orf551	AAC24230	88.34	523	0	*Cryphonectria parasitica*	551	LAGLIDADG
cd_rnl_orf486	YP_007507054	38.93	429	5.817e-81	*Ceratocystis cacaofunesta*	486	LAGLIDADG
cd_IN_orf333	1703265F	43.40	318	2.37845e-83	*cytochrome oxidase I intronic ORF*	333	LAGLIDADG
cd_IN_orf341	CAA38805	42.81	285	5.44616e-65	*Podospora anserina*	341	LAGLIDADG
cd_IN_orf126	CAA38805	42.81	285	5.44616e-65	*Podospora anserina*	126	LAGLIDADG
cd_nad1_i1_orf191	YP_008964988	74.59	181	1.9351e-86	*Annulohypoxylon stygium*	191	LAGLIDADG
cd_nad1_i2_orf185	NP_074958	59.32	118	1.92786e-32	*Podospora anserina*	185	LAGLIDADG
cd_nad1_i2_orf100	XP_007694002	74.16	89	1.20188e-38	*Bipolaris oryzae ATCC 44560*	100	LAGLIDADG
cd_nad1_i3_orf122	YP_009126720	75.41	122	8.41989e-55	*Neurospora crassa OR74A*	122	GIY-YIG
cd_nad4_i1_orf432	KIH86252	49.52	420	3.03061e-121	*Sporothrix brasiliensis 5110*	432	LAGLIDADG
**Query (Intron)**	**Top Hit Accession**	**% identity**	**Alignment length**	**E-value**	**Description**	**Query length**	
cp_IN_orf460	AMO66506	44.23	104	1.83211e-19	*Pyronema omphalodes*	460	LAGLIDADG
cp_cob_i1_orf356	1703265F	50.17	299	1.86329e-90	*Podospora anserina*	356	LAGLIDADG
cp_cob_i2_orf296	YP_009126715	74.43	305	8.7361e-156	*Neurospora crassa OR74A*	296	LAGLIDADG

cp_cob_i2_orf122	YP_009160660	45.24	42	0.000312056	*Hirsutella minnesotensis*	122	LAGLIDADG
cp_cob_i3_orf120	CAB72450	72.37	76	1.63084e-28	*Podospora curvicolla*	120	GIY-YIG
cp_cob_i3_orf201	XP_007693966	38.35	206	8.57381e-29	*Bipolaris oryzae ATCC 44560*	201	LAGLIDADG
cp_cox1_i1_orf824	YP_009029663	44.44	819	0	*Magnusiomyces ingens*	824	LAGLIDADG
cp_cox1_i2_orf446	AMO66533	44.67	394	2.18838e-92	*Pyronema omphalodes*	446	LAGLIDADG
cp_cox1_i3_orf782	NP_043734	48.50	765	0	*Allomyces macrogynus*	782	LAGLIDADG
cp_cox1_i4_orf393	AKQ53316	73.31	326	3.5926e-172	*Sclerotinia sclerotiorum 1980 UF-70*	393	GIY-YIG
cp_cox1_i5_orf434	YP_009072338	39.78	279	1.33149e-48	*Sclerotinia borealis*	434	GIY-YIG
cp_cox1_i5_orf173	YP_008964978	76.61	171	8.62356e-95	*Annulohypoxylon stygium*	173	LAGLIDADG
cp_cox1_i6_orf436	AKQ53319	76.06	426	0	*Sclerotinia sclerotiorum 1980 UF-70*	436	GIY-YIG
cp_cox1_i7_orf310	XP_002839373	62.79	258	1.61779e-96	*Tuber melanosporum Mel28*	310	LAGLIDADG
cp_cox1_i8_orf108	XP_014550036	62.26	106	9.62981e-32	*Bipolaris victoriae FI3*	108	GIY-YIG
cp_IN_orf113	KUI52547	72.38	105	8.06574e-46	*Valsa mali var. pyri*	113	LAGLIDADG
cp_IN_orf160	KUI52547	82.50	160	2.69467e-91	*Valsa mali var. pyri*	160	LAGLIDADG
cp_nad2_i1_orf473	AGN49028	70.98	386	0	*Botrytis cinerea B05.10*	473	LAGLIDADG
cp_nad2_i2_orf437	CCC14769	76.11	406	0	*Sordaria macrospora k-hell*	437	LAGLIDADG
cp_nad2_i3_orf335	XP_007335807	66.98	212	2.34515e-95	*Agaricus bisporus var. burnettii JB137-S8*	335	LAGLIDADG
cp_nad2_i4_orf788	NP_043734	44.46	767	0	*Allomyces macrogynus*	788	LAGLIDADG
cp_nad2_i5_orf433	CDL73098	64.76	437	0	*Fusarium pseudograminearum CS3427*	433	LAGLIDADG
cp_IN_orf414	YP_008757687	48.22	309	1.86954e-90	*Fusarium circinatum*	414	LAGLIDADG
cp_cox2_i1_orf326	YP_008854719	61.43	293	4.95172e-107	*Ganoderma sinense*	326	GIY-YIG
cp_cox2_i3_orf112	YP_009072388	86.00	100	2.36543e-49	*Sclerotinia borealis*	112	LAGLIDADG
cp_cox2_i3_orf282	CDL73554	34.62	286	3.2833e-48	*Fusarium culmorum CS7071*	282	LAGLIDADG
cp_cox2_i4_orf332	CDL73109	79.23	337	0	*Fusarium pseudograminearum CS3427*	332	GIY-YIG
cp_IN_orf259	XP_001802056	37.33	75	0.0948753	*Parastagonospora nodorum SN15*	259	LAGLIDADG

cp_IN_orf414	AMO66509	52.11	213	4.11136e-62	*Pyronema omphalodes*	414	LAGLIDADG
cp_IN_orf179	AGN49019	59.84	127	3.77332e-40	*Botrytis cinerea B05.10*	179	GI-YIG
cp_nad5_i1_orf540	NP_074944	64.76	542	0	*Podospora anserina*	540	LAGLIDADG
cp_nad5_i2orf168	AAO14105	100.00	168	5.23273e-115	*Cryphonectria parasitica*	168	LAGLIDADG
cp_nad5_i2_orf290	AAO14110	99.63	271	0	*Cryphonectria parasitica*	290	LAGLIDADG
cp_atp6_i1_orf812	AAF27656	100.00	764	0	*Cryphonectria parasitica*	812	LAGLIDADG
cp_atp6_i2_orf365	YP_008964995	72.07	376	0	*Annulohypoxylon stygium*	365	GIY-YIG
cp_atp6_i3_orf238	YP_001427393	71.86	231	2.81518e-109	*Parastagonospora nodorum SN15*	238	GIY-YIG
cp_atp6_i3_orf409	XP_003342404	63.30	376	2.06184e-152	*Sordaria macrospora k-hell*	409	GIY-YIG
cp_IN_orf232	KPP69029	32.84	67	7.24451	*Scleropages formosus*	232	LAGLIDADG
cp_rns_orf285	AAB84209	100.00	285	0	*Cryphonectria parasitica*	285	LAGLIDADG
cp_rns_orf509	AAB84210	98.55	415	0	*Cryphonectria parasitica*	509	LAGLIDADG
cp_rns_orf151	AAB84211	96.40	139	1.18862e-88	*Cryphonectria parasitica*	151	LAGLIDADG
cp_rns_orf416	AAB84212	100.00	416	0	*Cryphonectria parasitica*	416	LAGLIDADG
cp_IN_orf106	YP_008964996	50.51	99	5.86349e-18	*Annulohypoxylon stygium*	106	LAGLIDADG
cp_cox3_i2_orf318	AGN49000	63.91	302	2.57071e-129	*Botrytis cinerea B05.10*	318	LAGLIDADG
cp_cox3_i4_orf439	YP_009136869	65.44	408	2.99994e-178	*Fusarium culmorum*	439	LAGLIDADG
cp_IN_orf427	YP_009136815	40.48	331	3.80221e-64	*Fusarium culmorum*	427	LAGLIDADG
cp_IN_orf359	YP_009072328	44.31	255	2.9427e-47	*Sclerotinia borealis*	359	GIY-YIG
cp_IN_orf282	XP_007335271	44.29	280	2.77259e-68	*Agaricus bisporus var. burnettii JB137-S8*	282	LAGLIDADG
cp_IN_orf247	YP_009072317	71.07	242	4.28782e-121	*Sclerotinia borealis*	247	GIY-YIG
cp_rnl_orf320	CAB72450	45.19	239	2.21001e-44	*Podospora curvicolla*	320	GIY-YIG
cp_rnl_orf387	CCQ18563	43.11	283	5.47906e-67	*Ganoderma lucidum*	387	LAGLIDADG
cp_rnl_orf459	NP_074929	39.64	333	9.5764e-67	*Podospora anserina*	459	GIY-YIG
cp_rnl_orf246	AKM22622	66.29	175	7.87127e-77	*Cordyceps militaris*	246	LAGLIDADG
cp_rnl_orf194	GAO18872	55.37	177	2.87646e-54	*Ustilaginoidea virens*	194	LAGLIDADG
cp_rnl_orf851	AAC24230	100.00	851	0	*Cryphonectria parasitica*	851	LAGLIDADG

cp_rnl_orf268	YP_008964948	62.74	263	2.13196e-104	*Annulohypoxylon stygium*	268	LAGLIDADG
cp_nad1_i1_orf211	CCC14770	35.96	203	5.23672e-29	*Sordaria macrospora k-hell*	211	LAGLIDADG
cp_nad1_i1_orf131	YP_008964988	53.33	135	9.04755e-38	*Annulohypoxylon stygium*	131	LAGLIDADG
