# Supplementary material for: Intron Derived Size Polymorphism in the Mitochondrial Genomes of Closely Related Chrysoporthe Species
Source: PLoS One. 2016 Jun 6;11(6):e0156104. doi: 10.1371/journal.pone.0156104 (PMC4894602; doi:10.1371/journal.pone.0156104)
Supplement: S4 Fig — Intronic ORFs are depicted by yellow arrowed boxes on introns of genes where found and are labelled according to the gene and intron position. Intergenic ORFs are also depicted by yellow arrowed boxes and are labelled with “IN” prefix. (PDF) [file pone.0156104.s004.pdf]

S4 Fig. Physical maps of the mt genomes of *C. austroafricana*, *C. cubensis*, *C. deuterocubensis* and *C. parasitica* showing locations of all intronic and intergenic ORFs. Intronic ORFs are depicted by yellow arrowed boxes on introns of genes where found and are labelled according to the gene and intron position. Intergenic ORFs are also depicted by yellow arrowed boxes and are labelled with "IN" prefix.

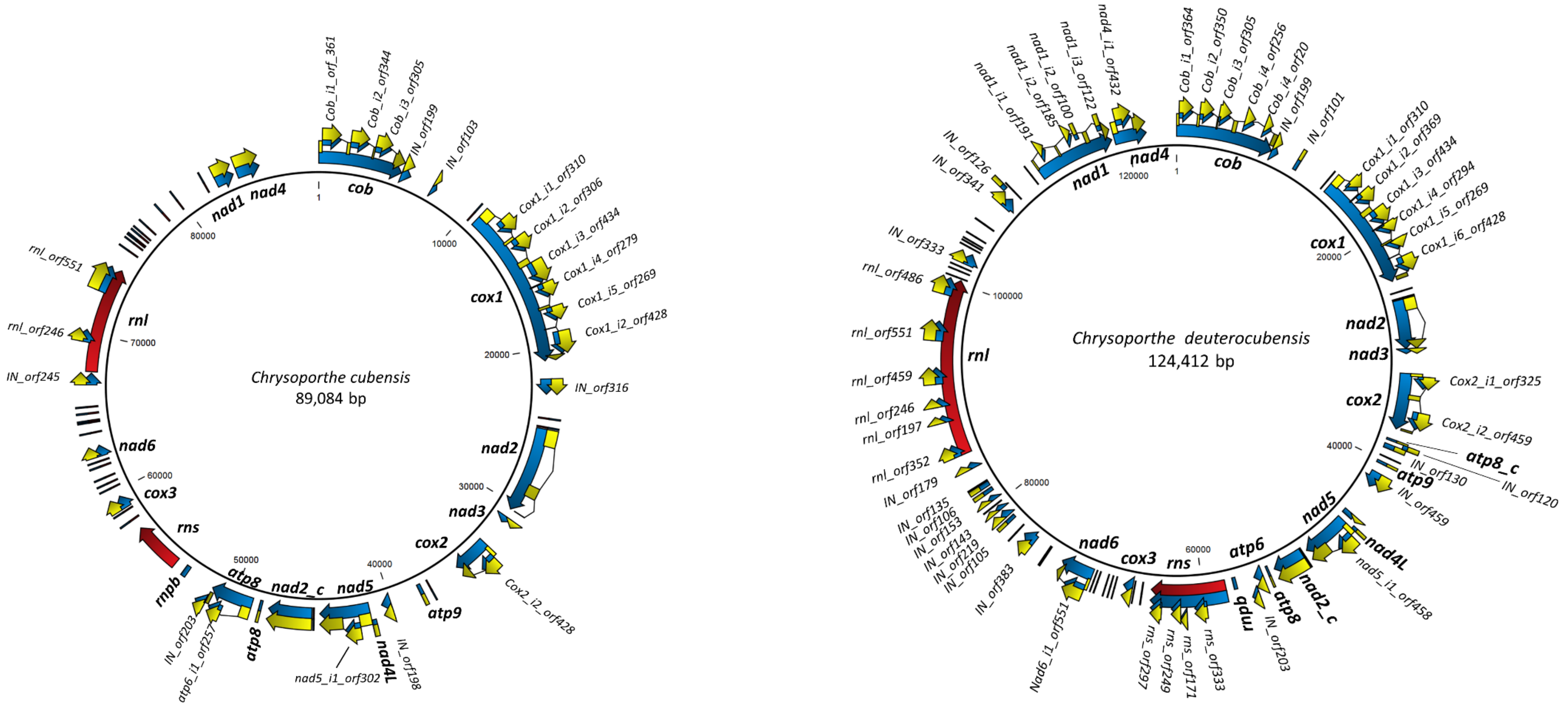

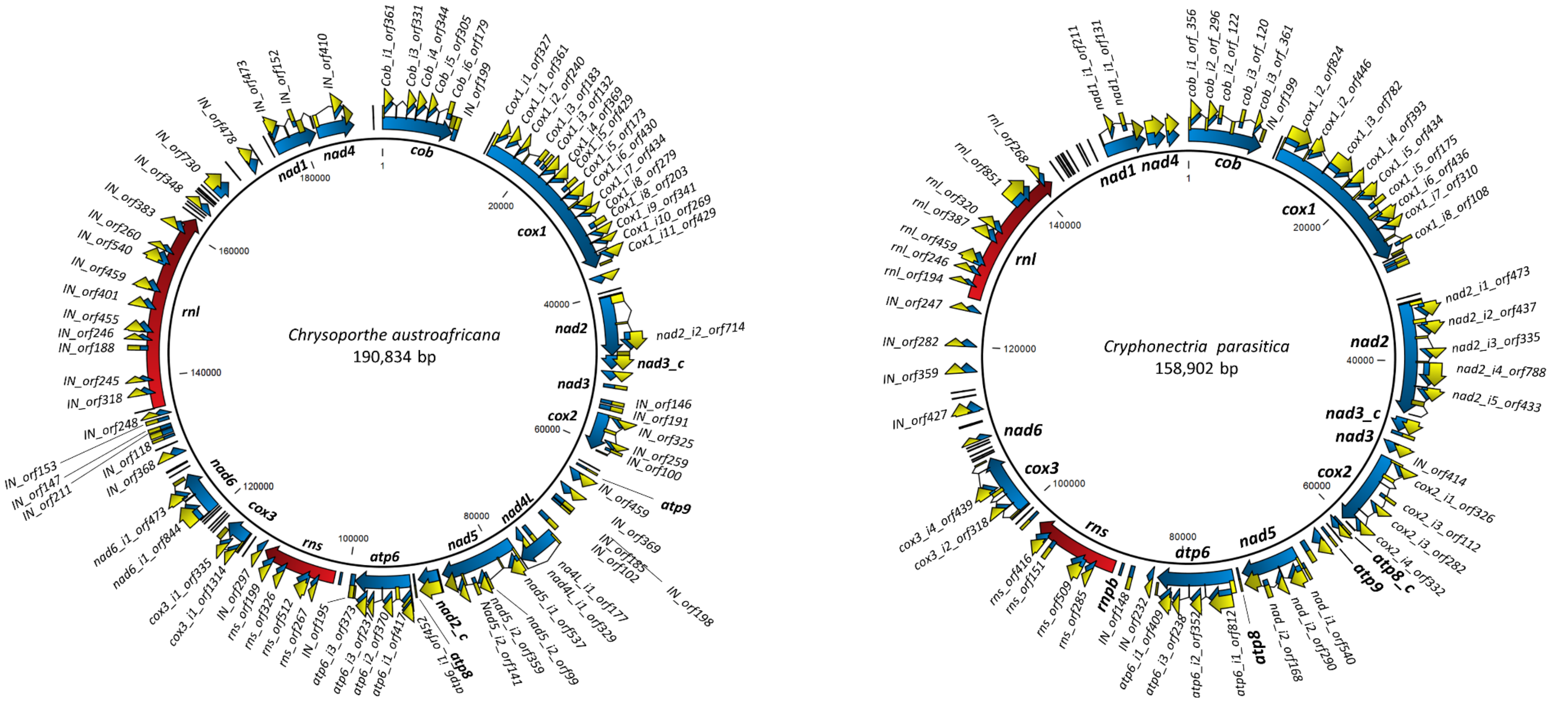
Chrysoporthe austroafricana
190,834 bp
cob
cox1
nad2
nad3_c
nad3
cox2
atp9
nad4L
nad5
nad2_c
atp8
atp6
rns
cox3
nad6
rnl
nad1
nad4
Cryphonectria parasitica
158,902 bp
nad1
nad4
cob
cox1
nad2
nad3_c
nad3
cox2
atp8_c
atp9
nad5
dtp6
atp8
rnpb
rns
cox3
nad6
rnl
